# Oral Microbiome–Derived Proteins in Brain Extracellular Vesicles Circulate and Tie to Specific Dysbiotic and Neuropathological Profiles in Age-Related Dementias

**DOI:** 10.1016/j.mcpro.2025.101464

**Published:** 2025-11-17

**Authors:** María Mulet, Jose Antonio Sánchez Milán, Cristina Lorca, María Fernández-Rhodes, Ana Adrados-Planell, María Consuelo Bejarano Castillo, Laura Saiz, María-Victoria Mateos-Moreno, Yoshiki Hase, Alex Mira, Alberto Rábano, Teodoro del Ser, Raj N. Kalaria, Anna Lagunas, Mònica Mir, Andrés Crespo, Josep Samitier, Xavier Gallart-Palau, Aida Serra

**Affiliations:** 1 Biomedical Research Institute of Lleida (IRBLLEIDA) - +Pec Proteomics Research Group (+PPRG) - Neuroscience Area – University Hospital Arnau de Vilanova (HUAV), Lleida, Spain; 2 Department of Medical Basic Sciences, University of Lleida (UdL), Lleida, Spain; 3 Department of Genomics and Health, FISABIO Foundation, Valencia, Spain; 4 Centro de Salud Argüelles, Servicio Madrileño de Salud, Madrid, Spain; 5 Alzheimer's Centre Reina Sofia-CIEN Foundation-ISCIII, Research Platforms, Madrid, Spain; 6 Department of Dental Clinical Specialties, Faculty of Dentistry, Complutense University of Madrid, Madrid, Spain; 7 Translational and Clinical Research Institute, Campus for Ageing and Vitality, Newcastle University, Newcastle, UK; 8 Networking Biomedical Research Center in Bioengineering, Biomaterials and Nanomedicine (CIBER-BBN), Barcelona, Spain; 9 Nanobioengineering Group, Institute for Bioengineering of Catalonia (IBEC) Barcelona Institute of Science and Technology (BIST), Barcelona, Spain; 10 Department of Electronics and Biomedical Engineering, University of Barcelona, Barcelona, Spain

**Keywords:** oral microbiome, mild cognitive impairment, Alzheimer’s disease, extracellular vesicles, vascular dementia

## Abstract

The involvement of the oral microbiome (OM) in the pathophysiology of Alzheimer's disease and vascular dementia has been recognized epidemiologically, but the molecular mechanisms remain elusive. In this study, we uncovered the presence of OM-derived proteins (OMdPs) in brain extracellular vesicles (bEVs) from post-mortem Alzheimer's disease and vascular dementia subjects using unbiased metaproteomics. OMdP circulation in blood EVs was also confirmed in an independent cohort. Our findings also reveal that specific OMdPs are present in bEVs, with their levels varying with disease progression. Peptidome-wide correlation analyses further explored their exchange dynamics and composition within bEVs. In addition, we validated the ability of OM-derived EVs to cross the blood–brain barrier using a blood–brain barrier–on-a-chip model, confirming a potential route for bacterial-derived molecules to reach the central nervous system. Bioinformatics-driven interaction analyses indicated that OMdPs engage with key neuropathological proteins, including amyloid-beta and tau, suggesting a novel mechanism linking dysbiotic OM to dementia. These results provide new insights into the role of the OM in neurodegeneration and highlight OMdPs as potential biomarkers and therapeutic targets.

## Highlights

• Relevant proteomic evidence of oral microbiome (OM)–derived proteins in brain extracellular vesicles (EVs) from dementia patients.
• Stage-specific modulation of OM-derived proteins identified across Alzheimer’s disease progression.
• *In silico* analysis reveals direct interaction of OM-derived proteins with amyloid and tau pathology–associated proteins.
• OM-derived proteins validated in plasma EVs from an independent Alzheimer’s disease cohort.
• OM-derived EVs demonstrated to cross the blood–brain barrier *in vitro*.

## Graphical Abstract

**Figure:**
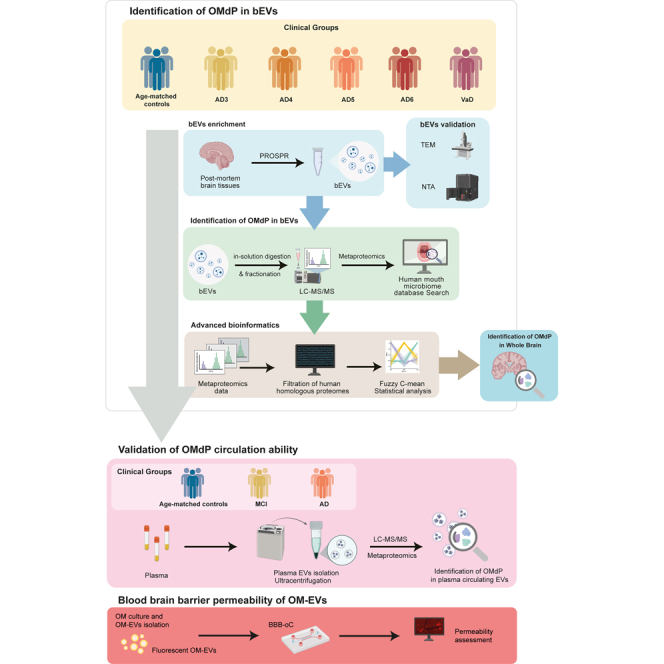

## In Brief

This study by Mulet *et al*. characterizes oral microbiome–derived proteins in brain extracellular vesicles from post-mortem samples of individuals with Alzheimer's disease and vascular dementia, revealing stage-specific alterations and molecular interactions with amyloid and tau pathology. Complementary validation in plasma and assessment of blood–brain barrier crossing using an *in vitro* chip model further underscore the contribution of dysbiosis to dementia progression, providing new insights into underlying neuropathological mechanisms.

Alzheimer’s disease (AD) and vascular dementia (VaD), the most common causes of dementia (1, 2), involve progressive damage of the central nervous system (CNS) cells, with a special incidence on neurons (3, 4). Several pathological mechanisms, such as chronic neuroinflammation, acute oxidative stress, neurovascular unit disintegration, and imbalanced cell homeostasis, have been centrally implicated in the neurodegenerative and vascular processes (4, 5, 6, 7, 8). It is remarkable, however, that most of the research performed to date to further understand the physiopathology of these dementing diseases has centered exclusively on the study of the host's genetic, metabolic, and protein dysfunctions (9, 10), neglecting the fact that mammals, and most specifically humans, are holobionts that only achieve systemic homeostatic functioning through the symbiotic action of multiple organisms and microbial agents (11, 12, 13).

The mammalian body hosts essential microbiota in the integumentary, reproductive, olfactory, and digestive systems (14). It is in the latter—particularly within the oral cavity—where one of the most densely populated and intensively linked to systemic human health microbiota resides (15, 16). Bidirectional communication and cross-regulation between CNS cells and agents of the oral and gut microbiotas have largely been hypothesized (17, 18, 19), and the ability of brain cells to sense microbiota status molecularly has been recently documented (20). However, the mechanisms underlying these interactions remain poorly understood. Similarly, the relationship between microbiota production of certain molecules through dysbiosis, including polysaccharides, and an increase in senile plaque burden in patients with AD has also been hypothesized (21, 22, 23). Several epidemiological studies also suggest existing links between oral health deterioration, neuropathology, and cognitive decline (24, 25, 26, 27). Furthermore, the presence of certain oral microorganisms in the brains of patients with dementia has been previously reported (28, 29). However, the paths by which these microorganisms or any of their potential products trespass the blood–brain barrier (BBB) throughout the course of AD and VaD are yet to be established.

It is widely known that intercellular communication within the CNS is actively mediated by vesicles; however, the involvement of extracellular vesicles (EVs) in some of these molecular exchanges and in bidirectional intercellular communication from and to the CNS has only been uncovered since the last decade (30, 31, 32, 33). EVs are tiny vesicles (50–2000 nm) formed by a lipid bilayer membrane that contains signaling lipids and transmembrane proteins and encloses a myriad of molecules, including proteins, metabolites, and genetic material (34, 35). These vesicles are made up, released, and taken up by almost all cells in all kingdoms of life (36, 37, 38, 39, 40). EVs show a proven ability to facilitate molecular interspecies exchange (41); and, as we have recently demonstrated, EVs of bacterial origin show outstanding ability to trespass the mammalian BBB following oral administration and remain in brain tissues further beyond 24 h (42).

Several authors, including us, have intensively contributed to improving the challenges that hinder the study of CNS-EVs by systems biology (31, 43) and to deciphering the potential neuropathological implications and biological marker abilities of brain EVs (bEVs) in dementias (44, 45, 46). However, matters such as whether EVs in the brain may contain molecules of bacterial origin linked to the oral microbiota and its dysbiosis, or whether these particular molecules can interact with the human proteome, remain, to the best of our knowledge, widely unresolved.

In this study, we demonstrate for the first time the presence of oral microbiome (OM)–derived proteins (OMdPs) in bEVs. We show how these molecules, linked to oral microorganisms, alter their presence and levels throughout the evolution of dementing diseases such as AD and VaD. We also analyze the potential exchange abilities of these proteins from bEVs to CNS cells. In addition, we implemented an *in silico* dynamic modeling to understand and predict whether these OMdPs have the chance to interact with specific AD-linked proteins, such as presenilins (PSEN) and amyloid β (Aβ) peptide. Finally, we demonstrate the circulating ability of OMdP in blood plasma EVs (pEVs) of an alternative and independent clinical cohort of patients suffering AD-related cognitive decline and the capacity of cultured OM-derived EVs (OM-EVs) to cross the BBB in the *in vitro* model of the human BBB assembled in a microfluidic device.

## Experimental Procedures

### Experimental Design and Statistical Rationale

**Fig. 1:**
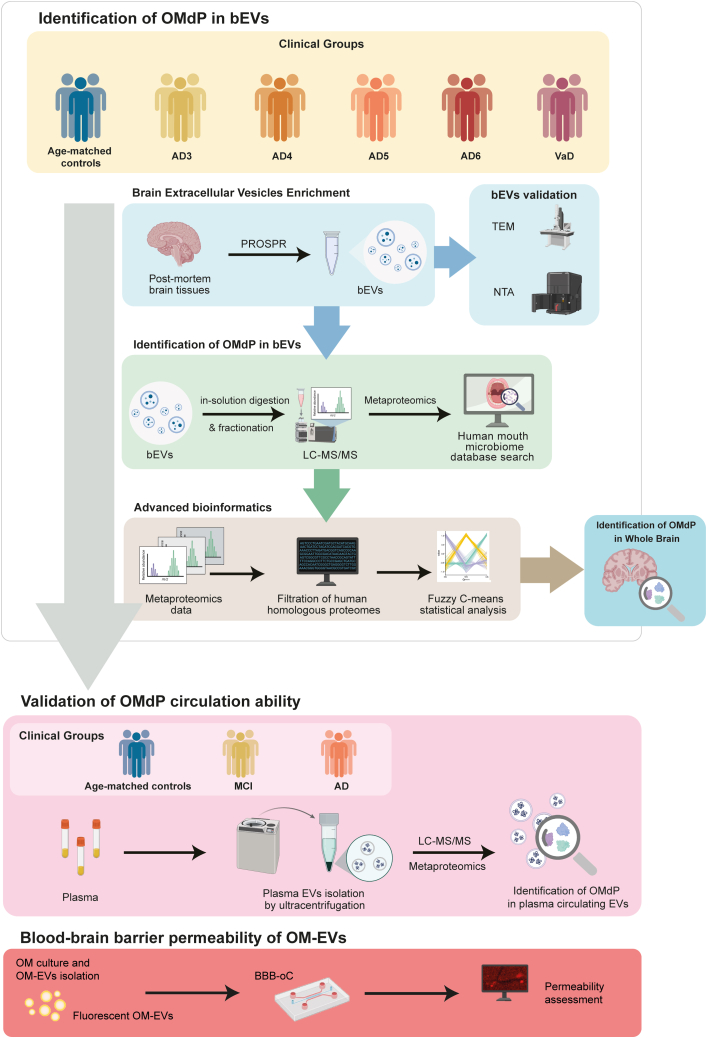
Illustrative diagram depicting the methodological approaches undertaken in this study to identify oral microbiome–derived proteins (OMdPs) in brain extracellular vesicles (bEVs) and validate their circulation ability in plasma.

This study was designed to explore the presence and potential role of OMdPs in dementia by combining discovery-driven proteomics in bEVs and validation of their circulation ability and potential biomarker capacity in pEVs. In addition, their presence was explored in whole brain (WB) tissues by tandem mass tag (TMT)–based proteomics. The experimental workflow is summarized in Figure 1. Briefly, bEVs were isolated from post-mortem brain tissue samples of neuropathologically characterized individuals (n = 18). Candidate OMdPs identified in bEVs were then evaluated in pEVs isolated from an independent plasma cohort of clinically diagnosed mild cognitive impairment (MCI), AD patients, and controls. For the pEV validation phase, 29 subjects were considered, including the proteomics characterization of pEVs from 10 subjects with AD, 10 subjects with MCI, and 9 age-matched controls. *Post hoc* power analysis indicated that this sample size (n = 20 per group) provides >80% power to detect large effect sizes (Cohen’s *d* = 1.0) at a significance level of α = 0.05. The brain proteomic study was designed as an exploratory discovery phase aimed at identifying OMdPs present in bEVs at different stages of AD. All sample extractions were conducted in a laminar-flow cabinet using sterile, 0.22-μm–filtered buffers and single-use sterile materials. The sample size was limited by the availability of post-mortem brain tissues from neuropathologically well-characterized individuals, including cases representing different Braak stages and disease progression levels. Findings from this exploratory brain analysis were used to select candidate OMdPs for subsequent validation in plasma samples in a bigger cohort.

All EV samples were processed individually, and proteomics was performed with biological and technical replicates to ensure data quality. Protein identifications were filtered using a false discovery rate (FDR) of <1%. For statistical analyses, adjusted *p* values were calculated using FDR <0.05 (*via* the Benjamini–Hochberg correction) to control for multiple comparisons and minimize false positives. Data quality and variability were assessed, and differential protein abundance analyses were complemented by *in silico* interaction and pathway enrichment analyses to support biological interpretation. In addition, to assess the permeability capacity of EVs derived from the OM, we used an organ-on-a-chip model of the BBB.

### Clinical Samples

In this study, human samples from a total of 47 subjects were analyzed. Post-mortem brain tissue samples (Brodmann area BA21) were obtained from 18 subjects within a post-mortem delay of 18.0 ± 6 h and an average age of 72.4 ± 8 years (Supplemental Table S1). Post-mortem brain tissue samples were obtained from individuals that showed at the time of autopsy early to advanced histological signs of AD or VaD and age-matched controls. Detailed information regarding gender, post-mortem delay, and age is included in Supplemental Table S1 (44). Age-matched controls referred to cognitively preserved aged subjects with no pathological burden of either AD or VaD or other related neurodegenerative conditions at the time of autopsy. All the cases in the AD and VaD groups exhibited amyloid plaques and neurofibrillary tangles. AD subjects were categorized according to the pathological Braak stage in early AD (Braak stage III; AD3) as clinical AD with mild symptoms of cognitive deterioration (44) and three groups of subjects that exhibited moderate to advanced clinical symptoms of dementia: intermediate AD (Braak stage IV; AD4), advanced AD (Braak stage V; AD5), and final stage AD (Braak stage VI; AD6). The clinical symptoms involved the spread of tau aggregates from the limbic regions to the neocortex, a fact associated with cognitive decline and AD symptomatology (45, 46). The VaD cases showed AD pathology mixed with significant cerebrovascular lesions, including small vessel disease primarily affecting the temporoparietal region as well as lacunar or gross infarcts. Three clinical replicates were included per condition, and each was analyzed independently in triplicate, unless specified otherwise. The post-mortem tissue samples were used for the characterization of bEV proteomes and WB proteomes, corresponding to brain tissue homogenates that were depleted of EVs. These samples were provided by the Harvard Brain Tissue Resource Center in Boston and the Newcastle Biobank in Newcastle, UK. Brain tissues were obtained at the time of autopsy and stored in liquid nitrogen at −150 °C until further processing. The BA21 tissues were cut into small pieces, and large blood vessels were removed. The tissues were then washed three times with 1X PBS for 30 min. Approval of the ethics committee for the study was obtained from the Spanish National Institute of Health (Instituto de Salud Carlos III) through Centro Alzheimer Fundación Reina Sofía-Fundación CIEN with reference: CEI PI 74_2021.

Proteomics data from plasma samples obtained from 29 subjects and previously generated by Nielsen *et al.* (47) were used to validate the circulation ability of OMdP. Briefly, plasma samples were obtained from the median cubital vein as previously described (47) from 10 patients with AD, 10 patients with MCI, and 9 age-matched controls. Neurological conditions were clinically verified as detailed (47). Briefly, AD diagnoses were based on the criteria from the National Institute of Neurological and Communicative Disorders and Stroke and the Alzheimer’s Disease and Related Disorders Association and the *International Classification of Diseases*, 10th Edition. MCI diagnosis was based on the Petersen criteria. Each plasma sample was independently analyzed in technical duplicate (*i.e.*, two LC–MS/MS injections per sample), except for one sample, which was injected only once because of technical issues. The use of human samples followed strictly the guidelines specified in the Declaration of Helsinki. All experimental procedures were carried out in accordance with the relevant institutional guidelines. Informed consent was obtained from the donors or their legal representatives.

### Brain Tissue Processing Prior to the Obtention of EVs

Brain tissue samples from each subject (∼80 mg) were homogenized using detergent-free homogenization buffer (consisting of 100 mM ammonium acetate supplemented with protease inhibitor cocktail tablets), as previously outlined (31). The tissue homogenizer bullet blender (Next Advance) was utilized for this process. Specifically, metallic beads with a diameter of 0.9 to 2.0 mm from Next Advance were washed three times with 1X PBS for 30 min before being combined with the brain tissue samples at a ratio of 1:1 (weight to weight). All steps in the process of obtaining EV brain fractions were carefully conducted at a temperature of 4 °C. Brain homogenization was performed four times, with each cycle comprising 300 μl of homogenization buffer and 5 min of homogenization. The initial two cycles were carried out at medium intensity, whereas the final two cycles were conducted at maximum intensity. After each cycle, the homogenate was centrifuged at 15,000*g* for 10 min, and the resulting supernatants were combined.

### Obtention of EVs

bEVs were enriched from detergent-free homogenates by protein organic solvent precipitation (in brain) (31). Briefly, brain homogenates were combined with four volumes of chilled acetone (at −20 °C) and then vortexed and centrifuged at 5000*g* for less than 1 min. The resulting supernatants containing hydrophobic EVs were subsequently concentrated to near dryness using a vacuum concentrator (Concentrator Plus; Eppendorf AG). Plasma EVs were enriched from 1 ml of plasma, as previously detailed (47), by performing two cycles of centrifugation at 100,000*g*, 1 h at 4 °C. EVs were then washed with PBS and resuspended in 20 μl PBS.

### In-Solution Digestion of EVs

Label-free in-solution digestion of protein organic solvent precipitation–isolated bEV was performed in accordance with previously established protocols (44). Briefly, the solubilization of bEVs was performed using 16 M urea and 100 mM ammonium bicarbonate buffer, which was then diluted twofold with HPLC water. The lysed EVs were subjected to reduction with 20 mM DTT at 30 °C for 3 h, followed by alkylation with 55 mM iodoacetamide for 1 h at room temperature in the dark. The samples were diluted to a urea concentration of less than 1 M with 50 mM ammonium bicarbonate. Subsequently, overnight trypsin digestion was carried out at 37 °C using a 1:20 enzyme-to-protein ratio (w/w) with sequencing-grade modified trypsin. The proteolytic digestion was halted by the addition of 0.5% formic acid (FA). The tryptic digested peptides from the EV fractions were desalted using a C18 Sep-Pak cartridge (Waters). Elution of bEV peptides was achieved using 1 ml of 75% acetonitrile (ACN) with 0.1% FA. The eluates were then dried in a vacuum concentrator and reconstituted in 200 μl of 0.02% ammonium hydroxide for subsequent fractionation by HPLC. For plasma EVs, commercial S-trap Micro Spin Columns (Protifi) were used following the manufacturer’s instructions (47).

### Proteome Extraction of Remaining WB Tissues

The subsequent processing of the brain tissue homogenates that were depleted of EVs involved the use of a homogenization buffer supplemented with 1% sodium deoxycholate (SDC), as previously detailed (48). This process was carried out to obtain the WB proteome. The homogenization process was carried out through five cycles at maximum intensity using the bullet blender homogenizer until no discernible pellet remained visible. The extracted proteins were then subjected to acetone precipitation by mixing brain tissue homogenates with chilled acetone for 3 h, followed by centrifugation at 20,000*g* for 10 min. The resulting supernatants were then discarded, and the precipitated WB proteomes were subjected to air-drying to eliminate excess acetone.

### In-Solution Tryptic Digestion and Isobaric Peptide Labeling of WB Proteomes

WB proteomes were solubilized in lysis buffer at pH  8.5, which included 1% SDC and 100 mM triethylammonium bicarbonate (TEAB), along with a complete protease inhibitor cocktail. Individual WB samples from subjects were combined to yield 200 μg protein/condition, following established procedures (49, 50). The bicinchoninic acid assay was used for protein quantification as previously detailed (51). WB proteomes were subjected to reduction using 10 mM Tris 2-carboxyethyl phosphine hydrochloride for 2 h at 55 °C, followed by alkylation with 55 mM iodoacetamide for 1 h at room temperature protected from the light. The SDC concentration was then reduced to 0.5% using 100 mM TEAB. Overnight trypsin digestion was conducted with sequencing-grade modified Promega trypsin at a 1:25 protein-to-enzyme ratio (w/w). The digestion process was stopped by adding a final concentration of 0.5% FA. SDC elimination from the digested samples was achieved through acidification, and peptides were retrieved from SDC precipitates as previously outlined (48). Tryptic peptides were dried in a vacuum concentrator before being reconstituted in 100 mM TEAB. The digested WB proteomes were then labeled using 6-plex isobaric tags (TMT, from ThermoScientific), following the manufacturer’s instructions. The TMT isobaric group labels were assigned as follows: 126 for age-matched controls (control), 127 for VaD, 128 for early AD (AD3), 129 for early intermediate AD (AD4), 130 for advanced stage AD (AD5), and 131 for final stage AD (AD6). Following labeling, peptides were desalted using a C-18 Sep-Pak 200 mg cartridge (Waters), and the eluates were dried thoroughly in the vacuum concentrator. The TMT-labeled peptide mixtures were then subjected to high-pH reversed-phase HPLC fractionation in order to reduce sample complexity prior to LC–MS/MS analysis. A total of 60 fractions were initially collected at 1-min intervals across a 60-min gradient. These fractions were then concatenated into pooled fractions noncontiguously.

### Simplification of bEV Proteomes by HPLC

Digested bEV proteomes were simplified by being subjected to HPLC fractionation as previously indicated (52, 53). To outline, the dried peptides were reconstituted in 200 μl of 10 mmol/l ammonium hydroxide in water (mobile phase A) and separated using an Xbridge BEH130 C18 column (3.5 μm, 4.6 × 250 mm; Waters) on a Shimadzu Prominence UFLC HPLC system (Shimadzu). Peptide intensities were monitored *via* UV at 280 nm throughout the process (54). Peptide separation occurred at a flow rate of 1 ml/min over a 60-min gradient: 0% to 5% B (consisting of 10 mmol/l ammonium hydroxide in ACN) for 3 min, 5% to 35% B for 40 min, 35% to 70% B for 12 min, and 70% to 100% for 5 min. Sixty fractions were collected (one per minute over a 60-min gradient) and subsequently concatenated into 20 final pooled fractions using a noncontiguous strategy (*i.e.*, pooling nonadjacent fractions across the chromatographic gradient, *e.g.*, 1, 11, 21, etc., into a single pool). The resulting pooled fractions were then completely dried in a vacuum concentrator.

### Liquid Chromatography MS/MS of EV Proteomes

Dried peptide fractions of bEV proteomes were reconstituted in mobile phase A (comprised of 3% ACN and 0.1% FA) prior to undergoing label-free shotgun proteomics analysis by means of LC–MS/MS. This process was carried out utilizing a Dionex UltiMate 3000 UHPLC system coupled with an Orbitrap Elite mass spectrometer (Thermo Fisher, Inc). The ion source utilized for spray generation was the EASY-Spray (Thermo Fisher Scientific, Inc), which operated at a voltage of 1.5 kV. Peptide separation was executed using a reverse-phase Acclaim PepMap RSL column (75 μm ID × 15 cm, 2-μm particle size; Thermo Scientific, Inc), maintained at a temperature of 35 °C and operating at a flow rate of 300 nl/min. A 60-min gradient was implemented for peptide separation, starting at 3% mobile phase B (90% ACN, 0.1% FA) for 1 min; followed by a linear increase to 35% over 47 min, an increase to 50% over 4 min, a linear ramp to 80% B over 0.1 min, an isocratic hold at 80% B for 78 s, a return to 3% over 0.1 min, and a final re-equilibration at 3% for 6.5 min. As a quality control (QC) measure, we injected a mixture of trypsin-digested standard proteins (albumin and ovalbumin) before and after each batch of samples to monitor the system’s performance. In addition, a pooled QC sample, consisting of equal aliquots from all experimental samples, was run periodically throughout the batches to further assess reproducibility and consistency. In this context, a coefficient of variation <20% was deemed appropriate (55). The Orbitrap Elite data acquisition was carried out in positive mode using Xcalibur 2.2 software (Thermo Fisher Scientific, Inc) alternating between full Fourier transform mass spectrometry (FT-MS; 350–2000 *m/z*, resolution 60,000, with 1 μscan per spectrum) and FT-MS/MS (150–2000 *m/z*, resolution 30,000, with 1 μscan per spectrum). Fragmentation of the 10 most intense precursors with charge ≥ + 2 and isolated within a 2 Da window was performed using high-energy collisional dissociation mode using 32% normalized collision energy. A threshold of 500 counts was enabled. For full FT-MS and FT-MS/MS, automatic gain control was set to 1 × 10^6^. LC–MS/MS of plasma EV proteomes was performed as detailed (47) using an Orbitrap Fusion Tribrid mass spectrometer doing a separation of peptides on an analytical EASY-Spray Column (50 mm × 75 μm, PepMap RSCL, C18, 2 mm, 100 Å; Thermo Scientific) in a 91 min gradient.

### LC–MS/MS of Isobaric-Labeled WB Proteomes

The reconstituted dried fractions of the WB isobaric-labeled proteome samples were analyzed using a Dionex Ultimate 3000 RSLCnano system coupled with a Q Exactive mass spectrometer (Thermo Fisher, Inc). The ionization was achieved using an EASY-Spray ion source (Thermo Fisher Scientific, Inc) operating at 1.5 kV. The peptide separation was carried out using a PepMap C18 column (3 μm, 100 Å; Thermo Fisher Scientific, Inc) maintained at 35 °C and operating at a flow rate of 300 nl/min. The separation of peptides occurred over a 60-min gradient with mobile phase A (0.1% FA in water) and mobile phase B (0.1% FA in 90% ACN) as follows: 3% to 30% B for 45 min, 30% to 50% B for 9 min, 50% to 60% B for 1 min, 60% B for 2 min, and finally maintained isocratically at 3% B for 3 min. The Q Exactive data acquisition was performed in positive ion mode using Xcalibur 3.0.63 software (Thermo Fisher Scientific, Inc), alternating between FT-MS (350–1600 *m/z* range, resolution of 70,000 at *m/z* 200, 1 μscan per spectrum) and FT-MS/MS (resolution 35,000) for the 10 most intense ions with charge ≥+2 and isolated within a 2 Da window. The ion fragmentation was achieved through high-energy collisional dissociation fragmentation mode using 28% normalized collision energy. A threshold of 500 counts was applied. For both full FT-MS and FT-MS/MS, automatic gain control was set to 5 × 10^6^ and 2 × 10^5^, respectively. As a QC measure, a mixture of trypsin-digested standard proteins (albumin and ovalbumin) was injected before and after each batch to monitor the system’s performance.

### Strain Culture

*Tannerella forsythia* strain American Type Culture Collection 43037 was thawed on Schaedler agar plates supplemented with sheep blood, hemin, and vitamin K1 (Thermo Scientific Oxoid) at 37 °C in an anaerobic chamber Whitley DG250 (Don Whitley Scientific), with an atmosphere of 85% N_2_, 10% CO_2_, 5% H_2_, during 3 days. *T. forsythia* culture was passaged into fresh minimal medium, consisting of Minimum salts M9 (500 ml/l), MgSO_4_ (2 ml/l), CaCl_2_ (0.1 ml/l), glucose 20% (20 ml/l), MEM Amino Acids Solution 50X (50 ml/l), MEM Nonessential Amino Acids Solution 100X (100 ml/l), and MEM Vitamin Solution 100X (20 ml/l). The minimal medium was used to avoid the presence of EVs from other organisms in our preparations. The initial inoculum amount for the culture was adjusted to achieve a cell density of absorbance of 0.5 at 600 nm and was incubated at 37 °C in an anaerobic chamber Whitley DG250 (Don Whitley Scientific), with an atmosphere of 85% N_2_, 10% CO_2_, and 5% H_2_. The culture was maintained in incubation for 24 h after the bacteria reached the stationary phase, with the stationary phase determined by measuring the absorbance at 600 nm every 2 h. The total incubation time was 32 h.

### Bacterial EV Isolation

The bacterial culture was centrifuged twice at 1900*g* for 20 min at 4 °C, followed by filtration through a 0.22-μm pore-size filter to remove the cellular fraction. Bacterial EVs were enriched from the culture by molecular weight cutoff filtration (42). Briefly, OM-EVs were enriched using 300 kDa Vivaspin 20 centrifugal filters (Sartorius) by centrifugation at 3000*g* for 15 min at 4 °C.

### BBB Permeability Assessment

To evaluate the ability of OM-EVs to cross the BBB, we utilized a BBB-on-a-chip (BBB-oC) model. The BBB-oC was obtained as detailed (56). Briefly, the main structural features of the BBB were reproduced in a microfluidic device, which contains a main chamber and two lateral microchannels, separated from the main chamber through microposts. Brain microvascular endothelial cells were seeded into one of the endothelial channels, whereas astrocytes and pericytes were cultured in the main chamber embedded in fibrin. The cells were maintained in culture up to 7 days, allowing the formation of a tight BBB-like monolayer, which was validated by transendothelial electrical resistance measurements. OM-EVs were labeled with Vybrant DiD Cell-Labeling Solution (Invitrogen), as previously described (42). Fluorescently labeled OM-EVs were introduced into the endothelial compartment, and their translocation across the BBB was analyzed using fluorescence imaging and quantitative assays.

### Morphometric Characterization of EVs

Three representative fractions of bEVs obtained from post-mortem brains of subjects diagnosed with early AD were randomly chosen for morphometric characterization by nanoparticle tracking analysis (NTA), as previously established (57). NTA analyses were carried out utilizing a Nanosight NS300 sCMOS instrument (Malvern Technologies) configured as follows: capture time of 60 s, camera level set to 4, slider shutter at 50, slider gain at 100, frames per second at 32.5, syringe pump speed at 100, total volume per sample at 1 ml, viscosity in the range of 0.906 to 0.910 cP, and temperature maintained at approximately 24 °C. In these analyses, no specific areas were designated as off-limits, enabling the random screening of unrestricted flowing samples. Three representative fractions of OM-EVs were selected at random and morphometrically characterized by dedicated flow cytometry. Dedicated flow cytometry experiments were performed on a Beckman Coulter Cytoflex SRT cell sorter (with side scatter detection in the violet laser). OM-EVs were labeled with Vybrant DiD Cell-Labeling Solution (Invitrogen), as previously described (42). Red fluorescence from the labeled OM-EVs was detected using the R 660/10 bandpass filter. Flow rate, fluorescence, and light scatter calibrations were performed on the day of the experiments. A buffer-only control (PBS) was measured with the same flow cytometer and acquisition settings as all other samples passed at 2 evt/s. Samples were passed at 300 to 500 evt/s during 5 min. We performed a trigger channel and threshold based on fluorescence. Fluorescence calibration was performed daily using Beckman Coulter Daily QC Calibration beads. Light scatter calibration was done with MegaMix-Plus Side Scatter Beads (Stago diagnostica).

### Ultrastructural Characterization of EVs

Three representative bEV samples were obtained from post-mortem brains of AD subjects, and OM-EVs were arbitrarily selected and prepared for transmission electron microscopy (TEM) analysis. The EV samples were applied onto Cu–Formvar–carbon grids and allowed to settle for 20 min at room temperature. Subsequently, the grids were washed with HPLC water, and the bEV preparations were fixed using 1% glutaraldehyde in PBS for 5 min. After fixation, the EVs were stained with uranyl oxalate for 5 min, followed by embedding in methyl cellulose–uranyl oxalate and air-drying for permanent preservation. Electron micrographs of the preparations were captured using a Jeol Jem 1010 electron microscope operated at 80 kV. The acquired ultrastructural images were then scale-calibrated, bileveled, and further analyzed using the open-source software ImageJ (National Institutes of Health).

### Next-Generation Proteomics of Circulating Blood Plasma EVs

In this study, we aimed to verify the circulating capabilities of OMdP in bEVs. To accomplish this, we sought out publicly accessible datasets from specialized repositories, such as ProteomeXchange, that had conducted analyses on blood plasma EVs in individuals with AD using LC–MS/MS next-generation discovery-driven proteomics. In this context, the term *“next-generation”* refers to unbiased data-dependent acquisition workflows performed on advanced hybrid mass spectrometers (*e.g.*, Orbitrap Elite), which provide superior mass accuracy, scan speed, and dynamic range compared with earlier quadrupole or time-of-flight instruments. Upon conducting a search for these criteria, we discovered the datasets that had been submitted by Nielsen *et al*. (47) with identifier PXD024216. The datasets in question comprise LC–MS/MS proteomics data of pEVs obtained from a total of 29 subjects, including 10 subjects diagnosed with AD, 10 subjects diagnosed with MCI, and 9 healthy age–matched controls. In this study, plasmatic EVs were obtained by double centrifugation at 100,000*g* for 1 h at 4 °C. Plasma EV proteomes were processed and digested using the commercial strategy S-Trap Micro Spin Column according to the manufacturer's instructions (Protifi). The label-free LC–MS/MS analysis of pEVs was carried out using a high-throughput instrument, the Orbitrap Fusion Tribrid mass spectrometer, as described previously (58), doing a separation of peptides on an analytical EASY-Spray Column (50 mm × 75 μm, PepMap RSCL, C18, 2 mm, 100 Å; Thermo Scientific, Inc) in a 91 min gradient.

### Bioinformatics and Statistical Analyses

#### MS Data Analysis

Analysis of proteomics raw data was performed using PEAKS Studio X Pro software (version 10.6; Bioinformatics Solutions). To control the FDR, a target-decoy strategy was employed. Specifically, a reverse decoy database was automatically generated by inverting the amino acid sequences of the target proteins and appended to the in-house database. Peptide-spectrum matches were scored against this combined target-decoy database, and FDR was estimated accordingly. A 1% FDR threshold was applied for protein identification in all samples. Trypsin was set as the proteolytic enzyme, and carbamidomethylation of Cys was set as a fixed modification. A precursor tolerance of 10 ppm and a fragment ion tolerance of 0.05 Da were allowed in the searches. Data were searched against an in-house–created database compiling all the available protein sequences in the Human Oral Microbiome Database (HOMD) (4,028,421 sequences, February 25, 2020). A database search of OMdP in WB proteome after isolation of bEVs was conducted with a restricted database containing the sequences of the OMdP identified in bEVs. In that search the TMT 6-plex tag masses were included as fixed modifications in the database search of the isobaric TMT labeling raw data, as previously detailed (44). Database search results were thoroughly exported into comma-separated values files. Label-free quantification of proteins was performed using spectral counting, in which the total number of MS/MS spectra assigned to peptides of each protein was used as a proxy for protein abundance. This approach was chosen for its robustness and reproducibility across multiple runs, particularly in fractionated data-dependent acquisition workflows, where it provides reliable semiquantitative estimates even in complex or low-abundance samples (59). Spectral counting is less sensitive to variations in chromatographic performance and does not require advanced feature alignment, making it suitable for multisample comparisons in our experimental setup using PEAKS Studio. Spectral counting was used as a semiquantitative measure of protein abundance. In addition, for validation purposes, a complementary analysis was performed using MaxQuant with restricted data search parameters (60). Protein intensities were calculated using the MaxLFQ algorithm, a reliable and robust label-free quantitation approach that provides improved accuracy and reproducibility across samples. To minimize potential biases because of peptide sharing across proteins or species in our multispecies searches, only unique peptides were considered for quantification. This ensured that each spectral count was derived from peptides unambiguously assigned to a single protein, thereby reducing crossattribution and improving the reliability of the spectral counting approach. For group-level analyses, spectral data were accounted for across all peptides assigned to the same protein and then averaged across replicates within each study group.

For validation purposes, representative MS/MS spectra of significant peptides were manually inspected to confirm sequence assignment. In addition, a restricted database search was performed using a reduced OM database that included only taxa and proteins confidently identified at least once in the initial full-database search. Spectral counts obtained from this restricted database were compared against those from the original search to assess consistency.

Cluster analysis based on label-free protein quantitation (total number of spectra detected for all peptides in each protein) was performed on the data from the bEV proteomes using the fuzzy c-mean algorithm in R software (version 4.2.1) (R Foundation for Statistical Computing), as previously detailed (61). Only proteins with a fuzzy partition coefficient ≥0.5 were considered. Lists of clustered proteins were further independently analyzed by parametric one-way ANOVA with Bonferroni correction for multiple comparisons, and statistical significance was set at corrected *p* ≤ 0.05, unless otherwise specified. Missing peptide intensities recorded as “not detected” were replaced with zero values prior to protein-level quantification. To validate these initial findings, two complementary validation analyses were conducted. First, spectral count data were subjected to left-censored imputation and subsequent log_2_ transformation prior to statistical testing. Second, peptide-level quantitation data obtained from MaxQuant processing were analyzed using the MaxLFQ algorithm, followed by left-censored imputation and log_2_ transformation. Consistent with the observation that many missing values in label-free proteomics arise from low-abundance peptides (62), this approach allowed imputing values near the detection limit prior to statistical re-evaluation. The resulting datasets were re-evaluated statistically to test the robustness and consistency of the original findings.

#### Identification of OM-Derived Peptides and Statistical Analyses

To accurately identify the tryptic-digested peptides derived from OMdP in bEVs present in the analyzed samples, we utilized a rigorous screening process to remove any human-homologous peptide. For that purpose, all possible isoleucine–leucine variants of each identified OMdP in bEV peptide were generated and compared against the human UniProt proteome database (79,703 sequences; June 27, 2022) using an in-house script created using Spyder software (Python Software Foundation). At the end, any peptide present in the human proteome was excluded from further analysis to ensure proper accuracy of the obtained results.

The identified proteins and peptides were then further analyzed by parametric one-way ANOVA with Bonferroni correction for multiple comparisons, and statistical significance was set at corrected *p* ≤ 0.05, unless otherwise specified. Statistical analyses were performed in GraphPad Prism, version 9.0.2 (GraphPad Software).

Multiple correlation analyses were performed in R by calculating the Pearson’s correlation coefficients between study groups at the protein level. A 95% confidence interval was used, and strong interaction between correlated variables was considered when *r* ≥ 0.70, whereas very strong interaction between correlated variables was considered when *r* ≥ 0.85. Significance of the interaction was established when *p* ≤ 0.05.

#### Oral Microbiome

The identification of OMdP-producing microorganisms was based on an in-house curated database, which was built using proteome data downloaded from the HOMD. Each protein in this database was linked to its corresponding bacterial species detailed in HOMD, ensuring that the specific producer organism was recorded and maintained in the dataset. The bacterial information was included in the name of each entry in the database, reflecting its original annotation in the HOMD. In addition, information regarding the natural habitat of microorganisms potentially producing the identified OMdPs in bEVs was sourced from HOMD. These data, along with pathogenicity information, were manually curated by reviewing relevant literature on their pathogenicity and association with human diseases available in PubMed. All references used for these classifications are provided in the corresponding supplemental data tables.

#### *In Silico* Molecular Dynamics

*In silico* docking simulations were carried out using the HDOCK software, version 2.2 (Huanglab) (63). Protein structures were obtained in Protein Data Bank format from the Research Collaboratory for Structural Bioinformatics (http://www.rcsb.org) or the AlphaFold database from the European Bioinformatics Institute (https://alphafold.ebi.ac.uk). The 10 most probable interaction models generated in each *in silico* simulation were carefully examined, and the one with higher confidence score was selected. From every docking simulation, the interaction in Protein Data Bank format was generated, along with a list of interacting residues and a confidence score (a confidence score >0.7 indicates that the two molecules are likely to bind (64)). Chignolin was used as a negative control for the docking simulation. The selection of human brain proteins linked to neuropathology was predicated on their common interactions with the human homologs of the OMdP in bEVs. Human homologous proteins of OMdP in bEVs were identified from UniProt, and a BLAST analysis was conducted to verify sequence homology. Functions of every human homologous protein of OMdP in bEVs were obtained from UniProt and GeneCards. Protein images were created and processed using PyMOL, version 3.0.2 (The PyMOL Molecular Graphics System, version 3.0.2; Schrödinger, LLC).

## Results

### Identification of Common Microbiome-Derived Proteomes in bEV Preparations

**Fig. 2:**
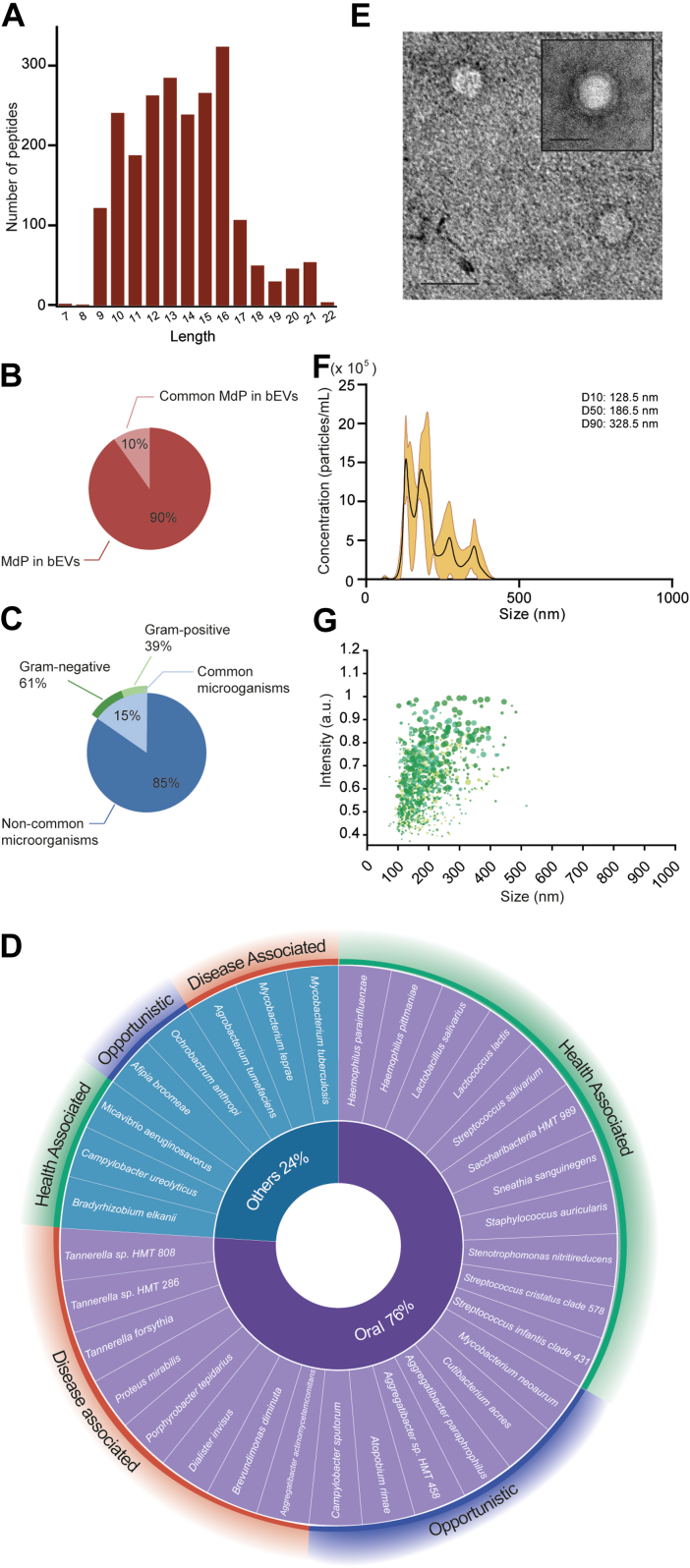
**Characterization of microbiome-derived proteomes (MdPs) in brain extracellular vesicles isolated from human post-mortem brain tissues considering all experimental conditions.***A*, peptide sequence length distribution of MdP in bEVs ranging from 7 to 22 amino acids detected by LC–MS/MS after tryptic digestion. *B*, percentage of MdP in bEVs regularly identified in all the subjects (common MdP in bEVs) and percentage of MdP in bEVs not identified in all the subjects (MdP in bEVs). *C*, sequences of the common MdP in bEVs are found in 15% of all the microorganisms’ proteomes potentially producing MdP in bEVs. Common microorganisms refer to the microorganisms that potentially produce the common MdP in bEVs. The rest of the microorganisms constitute the noncommon microorganisms. The classification of microorganisms into Gram-negative and Gram-positive is indicated by *dark green* and *light green* segments, respectively, within the subset of common MdP in bEV-producing microorganisms identified across all experimental conditions. *D*, list of microorganisms potentially producing the identified MdP in bEVs categorized according to their origin (oral: belonging to oral microbiome; or other: belonging to gut, respiratory, or skin microbiome [*inner pie chart*]) and pathogenicity (disease associated: microorganisms usually related to disease; opportunistic: they cause disease under specific conditions; and health associated: microorganisms usually related to health) (*outer circle*). *E*, representative transmission electron microscopy (TEM) micrographs of bEVs isolated by PROSPR from human post-mortem brain tissues. The scale bars represent 100 nm. Morphometric and quantitative characterization of bEVs assessed by nanoparticle tracking analysis (NTA). *F*, histograms showing the particle-size distribution. Standard deviation is represented as an *orange shadow*. *G*, histogram showing the scattering distributions of bEVs. Independent runs are shown in different *green tones*.

The comprehensive methodological process employed in this study has been illustrated in Figure 1. The primary objective was to assess the relationship between bEVs, which may function as intercellular and interspecies communication mediators, and specific microbiome-derived proteomes (MdP) in the brain. Using next-generation proteomics in conjunction with advanced systems biology, we observed the presence of MdP in all bEV preparations we analyzed. By adopting a bottom–up approach, we identified 536 MdP peptides, ranging in length from 7 to 22 residues, in bEVs from all analyzed subjects (Fig. 2*A*). These shared MdP peptides specifically correspond to 71 different proteins that were potentially attributed to 245 distinct microorganisms, as further detailed in Supplemental Dataset S1. In addition, 401 unique peptides that could be attributed to 219 specific microorganisms were also identified (Supplemental Dataset S1). Further analysis revealed that 10% of the total MdP in bEVs and 15% of the identified microorganisms that synthesize these proteins were common under all analyzed experimental conditions (Fig. 2, *B* and *C*, respectively). Of the microorganisms producing MdP in bEVs, 61% were Gram-negative, whereas 39% were Gram-positive (Fig. 2*C* and Supplemental Table S2). The quantitation of the proteins produced by common Gram-negative and Gram-positive bacteria along the groups is shown in Supplemental Figure S1. Our findings also revealed that 76% of these common microorganisms preferentially belong to the OM (Fig. 2*D* and Supplemental Table S3) and were linked to the seven specifically common OMdPs: 2,3-bisphosphoglycerate-dependent phosphoglycerate mutase, adenosylhomocysteinase (AHCY), ATP synthase subunit alpha, ATP synthase subunit beta (ATPD), chaperone protein DnaK, nucleoside diphosphate kinase, and triosephosphate isomerase (Fig. 2*D* and Supplemental Table S3). The remaining 24% of these specifically attributed microorganisms belonged to other realms, as illustrated in Figure 2*D* and Supplemental Table S3. Similarly, 32% of OMdP in bEVs identified in this study displayed disease-associated properties (Fig. 2*D* and Supplemental Table S3). Notable species in this category included *Tannerella forsythia*, *Proteus mirabilis*, *Dialister invisus*, and *Brevundimonas diminuta*, as depicted in Figure 2*D*. In addition, 24% of the OM microorganisms identified, which were linked to particular OMdP in bEVs, displayed opportunistic abilities. These include species and microorganism strains, such as *Aggregatibacter* sp. *HMT 458* or *Aggregatibacter paraphrophilus*, among others, as extensively detailed in Figure 2*D*.

In addition, to evaluate the relative abundance of the peptides detected in our dataset, we analyzed the distribution of peptide intensities across all samples. As shown in Supplemental Figure S2, 80% of the peptides displayed intensities below 6, indicating that most of the identified microbiome-derived peptides correspond to low-abundance features, consistent with the complex nature of bEV proteomes. To further validate the robustness of our peptide identifications, we conducted a manual inspection of the MS/MS spectra of the identified peptides. Only those peptides that were properly fragmented have been included, as demonstrated in Supplemental Figures S3-S5. Furthermore, a direct comparison of peptide intensities between the full and restricted database searches confirmed that all the spectral matches initially identified were consistently recognized through the use of a restricted database (Supplemental Table S4). Overall protein coverage remained stable, with a slight improvement observed for certain proteins (Supplemental Table S5), and some new peptide identifications were also encountered, which are listed in Supplemental Table S6. Collectively, these results substantiate the robustness of our initial database searches and peptide-level findings.

### Ultrastructural Characteristics and Nanoparticle Distribution of bEVs including OMdP

Subsequently, to explore the physicochemical traits of bEVs containing OMdP, we subjected various randomly chosen preparations of bEVs to thorough ultrastructural characterization. TEM micrographs revealed that these bEV preparations exhibited intact, regular bilayered spherical shapes within the commonly observed bEV diameter range (Fig. 2*E*), with most particles exhibiting diameters between 90 and 200 nm.

Further characterization of these bEV preparations was performed using NTA. This complementary technique, which measures hydrodynamic diameters of particles in suspension, revealed a broader size range than TEM, with particle diameters spanning approximately 40 to 400 nm (Fig. 2, *F* and *G*). The peak particle concentration was observed at around 150 nm (Fig. 2, *F* and *G*). In addition, the D10, D50, and D90 values were 128.5 nm, 185.5 nm, and 328.5 nm, respectively, reflecting sample heterogeneity and the presence of particles outside the 90 to 200 nm range.

### OMdP Levels in bEVs Undergo Specific Modulation Throughout AD Progression

**Fig. 3:**
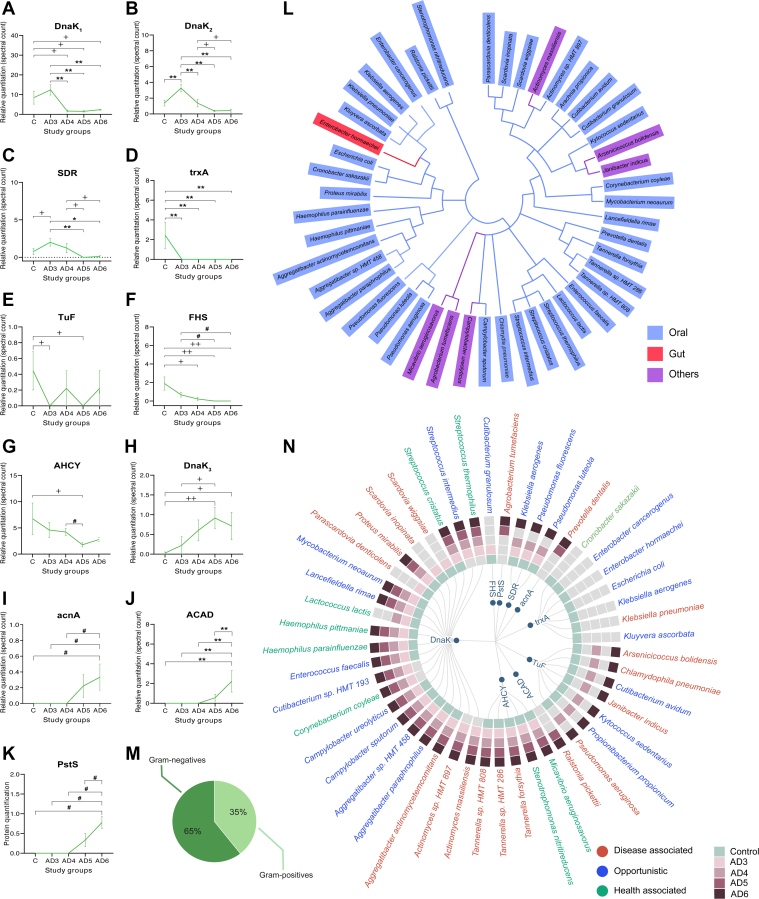
**Characterization of oral microbiome–derived proteins (OMdPs) in brain extracellular vesicles (bEVs) alongside the progression of Alzheimer’s disease (AD).***A*–*K*, LC–MS/MS quantitation of the significantly modulated OMdP in bEVs in post-mortem brain tissues of subjects at early AD (Braak stage AD3), intermediate AD (Braak stage AD4), advanced AD (Braak stage AD5), final stage AD (Braak stage AD6), and age-matched controls (*C*). Proteins are identified by their gene symbol, and protein quantitation is expressed as average sum of spectral count ± SEM. *L*, phylogenetic analysis of the microorganisms potentially producing the OMdP in bEVs significantly modulated in AD and its belonging habitat (oral: belonging to oral microbiome; gut: belonging to gut microbiome; or others: belonging to respiratory or skin microbiome). *M*, percentage of Gram-positive and Gram-negative in the OMdP in bEV-producing microorganisms. *N*, identification of OMdP in bEV-producing microorganisms in the experimental group based on the presence of unique peptide sequences (peptide sequences existing exclusively in a single protein). Gene symbol of OMdP in bEVs is indicated in the inner part of the diagram. All the microorganisms potentially producing every OMdP in bEVs are included. The presence of unique peptides in every experimental condition is identified with a color code. *Garnet tones* indicate the presence of unique peptides from this specific microorganism in the different stages of AD, and age-matched control condition is represented in *green*. *Gray* represents the absence of unique peptides for this microorganism in the specific condition. Microorganism names are colored according to their pathogenicity, as disease associated (*red*), opportunistic (*blue*), and health associated (*green*). Significant differences were assessed by two-way ANOVA with Bonferroni’s correction for multiple comparisons or nonparametric one-way ANOVA as indicated: ∗indicates significant differences at *p* ≤ 0.05, ∗∗indicates significant differences at *p* ≤ 0.01, + indicates significant differences determined by ANOVA with Fisher’s least significant difference (LSD) test *p* ≤ 0.05, ++ indicates significant differences determined by ANOVA with Fisher’s LSD *p* ≤ 0.01, and # indicates significant differences determined by nonparametric one-way ANOVA on ranks *p* ≤ 0.05.

Subsequently, our objective was to assess whether MdP exhibit alterations in the presence and abundance levels in bEVs alongside the progression of AD. To accomplish this, we performed a clustering analysis and sequential statistical analyses and found that a specific group of MdP in bEVs showed statistically significant differences during both early and subsequent advanced stages of AD, as demonstrated in Figure 3, *A*–*K*. In general, every MdP fitted within a specific cluster, except DnaK, which linked to different potential microorganisms of origin, displayed distinct modulation patterns alongside the AD progression based on abundance. Thus, four different DnaK isoforms were considered in subsequent analysis according to their disease modulation indicated by subscript numbers (DnaK_1_ to DnaK_4_) as detailed in Supplemental Table S7. Specifically, the following MdP in bEV heat shock proteins (HSPs; DnaK_1_ and DnaK_2_) and short-chain dehydrogenase (SDR) demonstrated a significant increase in early AD (Fig. 3, *A*–*C*). In contrast, MdP in bEV nerve growth factor-like (trxA) and elongation factor (TuF) showed a significant decrease in this preclinical phase of AD (Fig. 3, *D* and *E*). Several of these microorganism proteins were associated with samples from AD patients exhibiting increased disease severity, as indicated by specific downtrend and uptrend patterns in bEVs, including formate tetrahydrofolate ligase (FHS), AHCY (Fig. 3, *F* and *G*), and bacterial HSP (DnaK_3_), aconitate hydratase A (acnA), acyl-CoA dehydrogenase (ACAD), and phosphate-binding protein (PstS) (Fig. 3, *H*–*K*). The robustness of these results was further confirmed through validation analyses based on left-censored imputation and MaxLFQ quantitation, as summarized in Supplemental Tables S8 and S9.

Further screening revealed that these 11 formerly referred MdPs in bEVs were found significantly dysregulated, linked to the progression of AD, and found specifically by 46 specific microorganisms (Fig. 3*L* and Supplemental Table S10). The origin of these microorganisms was classified as from the OM, with over 84% belonging to this category, whereas less than 3% belonged to the gut microbiota and the remaining 13% belonged to other environments (Fig. 3*L* and Supplemental Table S10). Moreover, we found that 65% of these microorganisms were gram-negative bacteria, whereas only 35% were Gram-positive (Fig. 3*M* and Supplemental Table S11). The protein quantitation of the OMdP in bEVs that were significantly dysregulated during AD potentially produced by Gram-negative and Gram-positive bacteria is shown in Supplemental Figure S6. The results of these analyses demonstrated that approximately 37% of these microorganisms display disease-associated properties, whereas 44% are considered opportunistic, and 19% exhibit health-promoting characteristics (Fig. 3*N*). Furthermore, these analyses uncovered the presence of particular OMdP in bEVs from health-promoting microorganisms in the bEVs of age-matched controls and early AD subjects, as determined by the identification of unique microorganism-associated peptides (Fig. 3*N*). These microorganisms include *Cronobacter sakazakii* and *Cutibacterium granulosum* (Fig. 3*N*). In addition, we found specific OMdP in bEVs derived from disease-associated microorganisms in the bEVs of AD patients, which were not present in age-matched controls (Fig. 3*N*). These AD-linked disease-associated microorganisms include *Prevotella dentalis*, *Ralstonia picketti*, *Actinomyces massiliensis*, *Actinomyces* sp. *HMT 897*, and *Agrobacterium tumefaciens*.

### Specific OMdP Levels in bEVs Are Differentially Linked to VaD

**Fig. 4:**
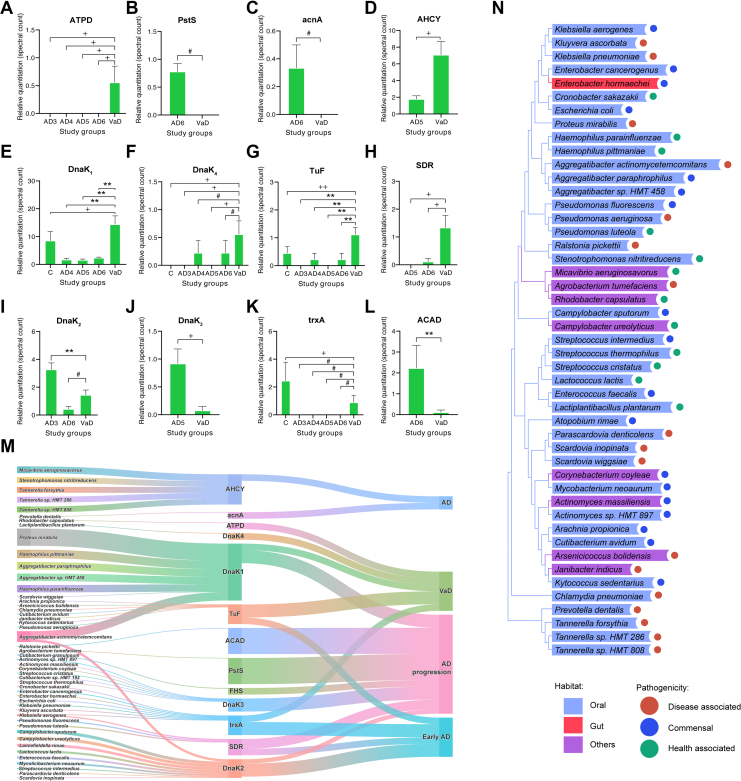
**Characterization of oral microbiome–derived proteins (OMdPs) in brain extracellular vesicles (bEVs) in vascular dementia (VaD).***A*–*L*, LC–MS/MS quantitation of the significantly modulated OMdP in bEVs in post-mortem brain tissues of subjects suffering from VaD compared with early Alzheimer’s disease (AD) (AD3), intermediate AD (AD4), advanced AD (AD5), final stage AD (AD6), and age-matched controls (*C*). Only conditions that had shown significant differences comparing to VaD are displayed. Proteins are identified by their gene symbol. Protein quantitation is expressed as average sum of spectral count ± SEM. *M*, Sankey diagram representing the connection between OMdP in bEV-producing microorganisms, OMdP in bEVs, and respective dementia-related categories, including the following categories: AD for OMdP in bEVs significatively modulated or only present in AD groups without an specific tendency toward the early stage of final stage of the AD progression, VaD for OMdP in bEVs significatively modulated or only present in VaD, AD progression for OMdP in bEVs that are significantly modulated alongside the AD progression, and early AD for those OMdP in bEVs significantly modulated or only present in early AD. *N*, phylogenetic analysis of the microorganisms potentially producing the OMdP in bEVs significantly modulated in VaD, its pathogenicity (disease associated, opportunistic, or health associated) and its belonging habitat (oral: belonging to oral microbiome; gut: belonging to gut microbiome; or others: belonging to respiratory or skin microbiome). Significant differences were assessed by two-way ANOVA with Bonferroni's correction for multiple comparisons or nonparametric one-way ANOVA as indicated: ∗indicates significant differences at *p* ≤ 0.05, ∗∗indicates significant differences at *p* ≤ 0.01, + indicates significant differences determined by ANOVA with Fisher’s least significant difference (LSD) test *p* ≤ 0.05, ++ indicates significant differences determined by ANOVA with Fisher’s LSD *p* ≤ 0.01, and # indicates significant differences determined by nonparametric one-way ANOVA on ranks *p* ≤ 0.05.

We subsequently examined whether OMdP in bEVs of individuals with VaD exhibit consistent results and/or specific differences in comparison to the observations made during the analysis of these proteins and microorganisms in AD. The majority of OMdP in bEVs identified as differentially regulated in the previously analyzed AD stages exhibited significant differences when compared with VaD, as demonstrated in Figure 4, *A*–*L*. Specifically, the bacterial protein ATPD was exclusively identified in the bEVs of subjects with VaD (Fig. 4*A*), whereas the bacterial PstS (Fig. 4*B*) and acnA (Fig. 4*C*) were not present in the bEVs of VaD subjects. Several OMdP in bEVs were found to be upregulated in the bEVs of VaD subjects compared with those from one or more of the analyzed AD stages. These included AHCY, the chaperones DnaK_1_ and DnaK_4_, TuF, and 2-keto-3-deoxy-l-fuconate dehydrogenase (SDR) (Fig. 4, *D*–*H*). In addition, several OMdPs were found to be downregulated in the bEVs of VaD compared with AD, including the chaperones DnaK_2_ and DnaK_3_, thioredoxin (trxA), and the dehydrogenase (ACAD) (Fig. 4, *I*–*L*). The consistency of these findings was corroborated by additional validation analyses applying left-censored imputation and MaxLFQ-based quantitation, as detailed in Supplemental Tables S8 and S9.

Our study aimed to subsequently determine any potential relationship that may exist between the proteins identified with specific profiles linked to VaD with potential OM microorganisms and dementia stages, as depicted in Figure 4*M*. The results of these analyses suggested that some organisms from the OM, such as *Rhodobacter capsulatus*, might be specifically associated with VaD, whereas other organisms, including *Chlamydia pneumoniae*, *Kytococcus sedentarius*, *Klebsiella pneumoniae*, and *Cronobacter sakazakii*, among others, may be linked to both VaD and early AD. However, no clear association was established between VaD and clinical AD at the OM level, as illustrated in Figure 3*M*. Furthermore, several microorganisms that may be responsible for producing the OMdP in bEVs associated with VaD were identified as specific to the OM and exhibiting opportunistic or health-associated properties (Fig. 4*N*).

### The Presence of OMdP in WB Proteomes Is Linked to Their Levels in bEVs

**Fig. 5:**
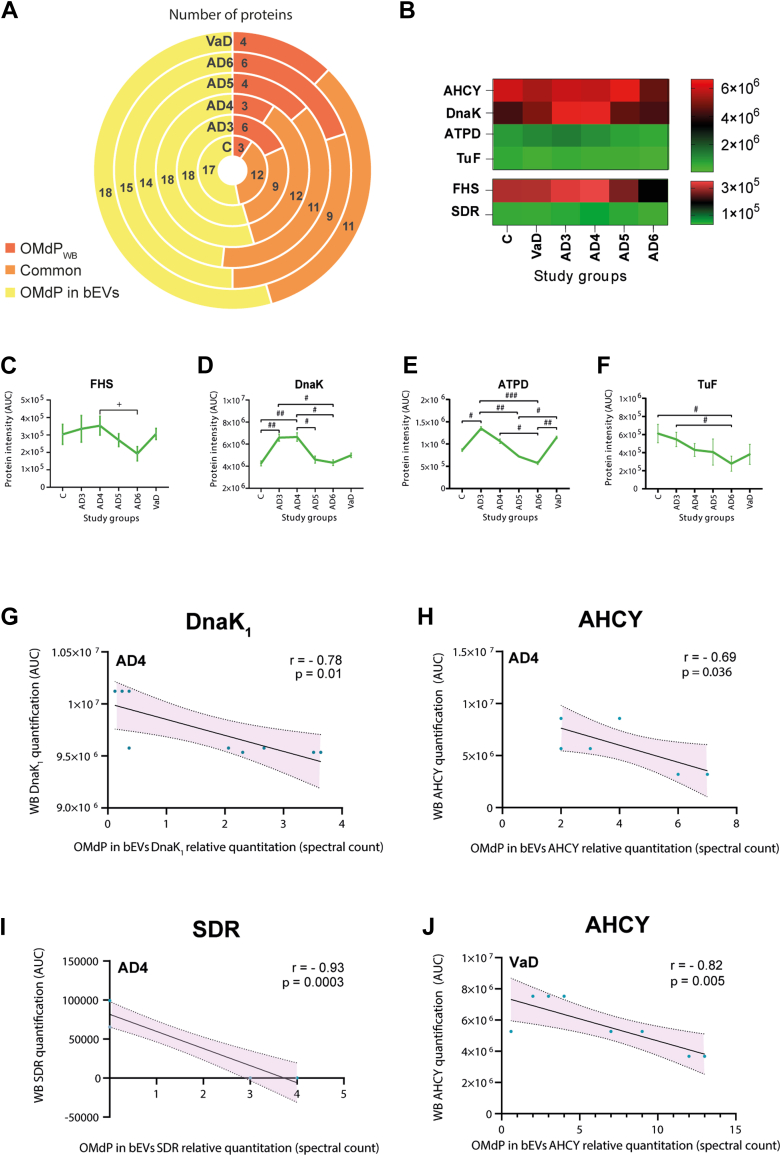
**Potential exchange ability of oral microbiome–derived proteins (OMdPs) between brain extracellular vesicles (bEVs) and brain tissues in Alzheimer’s disease (AD) and vascular dementia (VaD).***A*, comparative analysis of the presence of OMdP exclusively identified in bEVs (OMdP in bEVs), OMdP exclusively identified in the remaining whole brain (WB) proteome after isolation of bEVs (OMdP in WB) and identification of OMdP commonly present in both fractions bEVs and WB (common) in early AD (AD3), intermediate AD (AD4), advanced AD (AD5), final stage AD (AD6), VaD, and age-matched controls (*C*). Numbers indicate protein count. *B*, LC–MS/MS quantitation of OMdP in WB that displayed significant differences between groups in bEVs. Proteins are identified by their gene symbol. *Red and green colors* in the heatmap represent higher and lower intensity, respectively. *C*–*F*, LC–MS/MS quantification of OMdP in WB, expressed as area under the curve (AUC), which displayed significant differences between some of the analyzed groups. Average distribution and SEM are represented. +Significant differences determined by ANOVA with Fisher’s least significant difference (LSD) *p* ≤ 0.05 test. Significant differences determined by nonparametric one-way ANOVA on ranks *p* ≤ 0.05. Significant differences determined by nonparametric one-way ANOVA on ranks *p* ≤ 0.01. Significant differences determined by nonparametric one-way ANOVA on ranks *p* ≤ 0.001. *G*–*J*, correlation between the levels of common OMdP detected in bEVs and WB. Only significant correlations with *p* ≤ 0.05 are displayed. *Red shadow* shows the 95% confidence score.

The analysis of the presence and levels of OMdP in complementary brain tissues was carried out by performing next-generation proteomics of the remaining WB tissues after isolating bEVs (Supplemental Dataset S2). The study revealed that numerous OMdPs previously discovered in bEVs were also present in the examined WB excluding bEVs (Fig. 5*A*). In particular, the OMdP in bEVs: AHCY, DnaK, ATPD, TuF, FHS, and SDR, which were previously identified in bEVs and linked to AD and VaD, were also identified in WB (Fig. 5*B*). Analysis of these proteins in these complementary brain tissues revealed significant differences between dementia groups (AD and VaD) involving four of these proteins: FHS, DnaK, ATPD, and Tuf (Fig. 5, *C*–*F*). The plots of the proteins that did not show significant differences in WB portions are included in Supplemental Figure S7.

We subsequently undertook an extensive correlation study to determine whether any interaction existed between the levels of OMdP in bEVs and WB by analyzing the proteins commonly found in these brain vesicles and corresponding tissues, excluding bEVs. This study demonstrated that several of the dementia-modulated OMdP in bEVs exhibited significantly robust negative correlations between their levels in bEVs and their levels in WB (Fig. 5, *G*–*J*). In particular, significantly associated levels between the brain parenchyma and bEVs were identified in clinical cases of AD involving the OMdP DnaK_1_, AHCY, and SDR, as illustrated in Figure 5, *G*–*I*. In addition, a significant and statistically robust negative correlation was also observed for the OMdP AHCY in cases of VaD (Fig. 5*J*).

### OMdPs Display Docking Abilities With Key AD-Associated Proteins

**Fig. 6:**
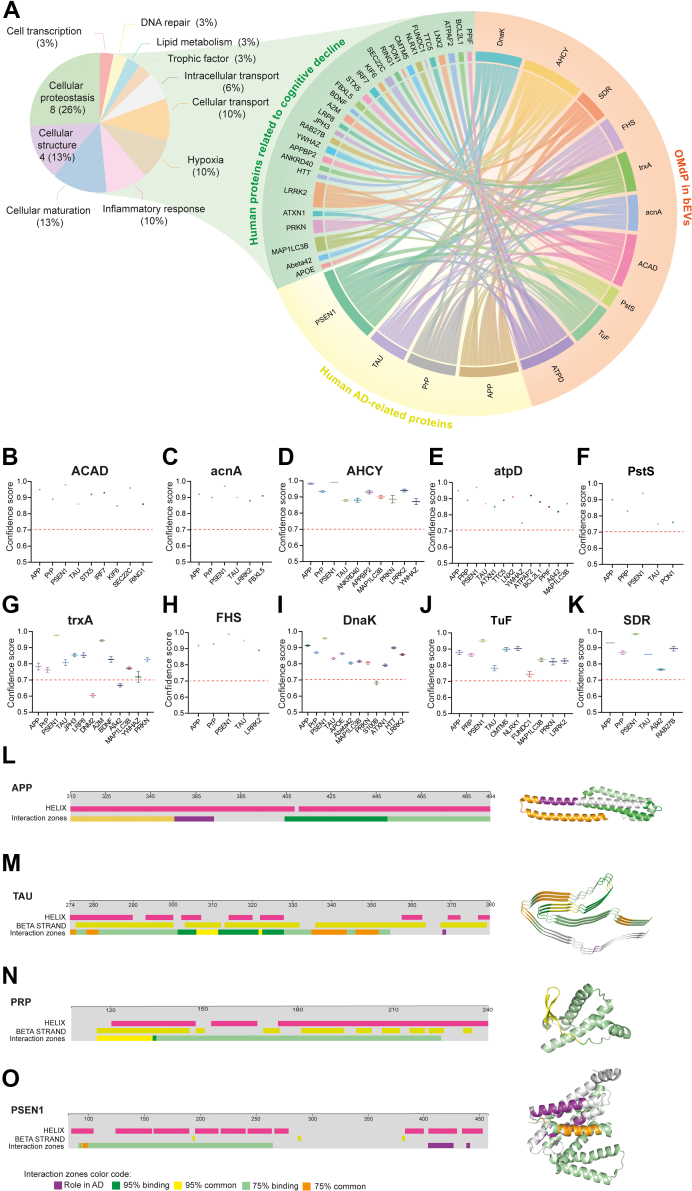
**Prediction of the interaction between oral microbiome–derived proteins (OMdPs) in brain extracellular vesicles (bEVs) and human proteins associated with cognitive decline and the neuropathology of human dementias.***A*, chord diagram presenting the OMdP in bEVs significantly modulated in Alzheimer’s disease (AD) (*orange*) related to human proteins associated with AD (*yellow*) and cognitive decline (*green*). The association of proteins is based on *in silico* docking simulations. The proteins associated to cognitive decline were manually screened based on scientific literature describing their association with the human homolog of the OMdP in bEV proteins. Only proteins with confidence score >0.7 are displayed. The functional analysis of the proteins associated to mental disorders with capacity to interact with OMdP in bEVs is displayed in the pie chart located on the *left*. *B*–*K*, graphical representation of the docking confidence scores obtained in the docking simulations between OMdP in bEVs and human proteins related to AD and cognitive decline. Docking simulations were performed using all the available Protein Data Banks (PDBs) of the OMdP in bEVs of every identified OMdP in bEV-producing microorganism. Average scores ± SEM is represented, except when only one docking simulation was performed. *Dashed red line* represents the 0.7 threshold. *L*–*O*, AD-related protein structure and regions involved in the interaction with OMdP in bEVs. For the proteins, amyloid precursor protein (APP; PDB code: 5BUO), microtubule-associated protein TAU (TAU; PDB code: 6VH7), presenilin 1 (PSEN1; PDB code: 7D8X), and prion protein (PRP; PDB code: 4KML), the linear representation of the sequence, and the 3-dimensional structure are displayed. The protein regions involved in the interaction with OMdP in bEVs are indicated with color according to the chance to participate in the interaction with OMdP in bEVs. Protein regions participating in ≥95% or 75% of the interactions with the significantly modulated OMdP in bEVs are represented in *dark green* and *light green*, respectively. Protein regions documented to play a role in the pathophysiology of AD participating in ≥95% or 75% of the interactions are displayed in *yellow* or *orange*, respectively*. Purple areas* represent the documented protein areas that are involved in AD neuropathology based on scientific literature (88, 89, 90, 91, 92, 93, 94, 95).

To assess the influence of molecular interactions involving OMdP in bEVs on the progression of AD, we examined their capacity to interact with human proteins that are associated with brain dementia. Specifically, we investigated the ability of dementia-modulated OMdP in bEVs to interact with proteins that are traditionally linked to AD neuropathology, such as amyloid precursor protein (APP), prion protein (PrP), tau protein (TAU), and PSEN1, as depicted in Figure 6*A*. In addition, we evaluated the capacity of dementia-modulated OMdP in bEVs to interact with specific human proteins that are related to cognitive decline by referencing the interaction capabilities demonstrated by their corresponding human homologs. Through this analysis, we identified 30 potential protein interactors that are associated with cognitive decline–related neuropathological factors (as shown in Fig. 6*A*, Supplemental Tables S12 and S13), such as brain-derived neurotrophic factor, Aβ42, APOE, PPIF, and ATPF2, among others. Additional analysis of this subset of proteins indicates that the majority of cognitive decline–associated proteins with homologous OMdP in bEVs are mainly connected to cellular proteostasis, comprising approximately 26% of the overall outcomes. Moreover, a considerable number of these proteins are associated with cellular structure, accounting for about 13% of the total (Fig. 6*A*).

*In silico* docking simulations were conducted to specifically evaluate the interactions between OMdP in bEVs and proteins associated with AD neuropathology, as well as with cognitive decline (Fig. 6, *B*–*K* and Supplemental Tables S14–S23). Significant dockings (with confidence scores greater than 0.7) (51, 64) were identified in these experiments involving OMdP in bEVs with several dementia-related proteins, including AD-neuropathological markers, such as APP, PrP, TAU, and PSEN1 (Fig. 6, *B*–*K*). The specific OMdPs in bEVs, SDR, ATPD, trxA, and DnaK were found to have the ability to dock with the neurotoxic AD peptide Aβ42 (Fig. 6, *E*, G, *I*, and *K*). The OMdP in bEVs trxA displayed the capacity to dock with crucial trophic factors, such as the brain-derived neurotrophic factor (Fig. 6*G*). To provide a control, *in silico* docking simulations with the randomly selected noninteracting protein (chignolin) were also conducted, and the resulting negative interactions are showcased in Supplemental Figure S8.

Further investigation was carried out to pinpoint the particular areas of the AD-neuropathological markers that interact with the previously identified OMdP in bEVs (Fig. 6, *L*–*O*). Notably, these studies demonstrated that OMdP in bEVs possess the ability to interact with regions of human proteins that are directly involved in proteinopathy and neurotoxicity in AD neuropathology in 95% of the simulated cases involving the proteins APP, TAU, and PrP (Fig. 6, *L*–*N*), whereas in 75% of the simulated cases involving PSEN1 (Fig. 6*O*).

### OMdP in bEV Fractions Associate With AD Markers at the Peptide Level

**Fig. 7:**
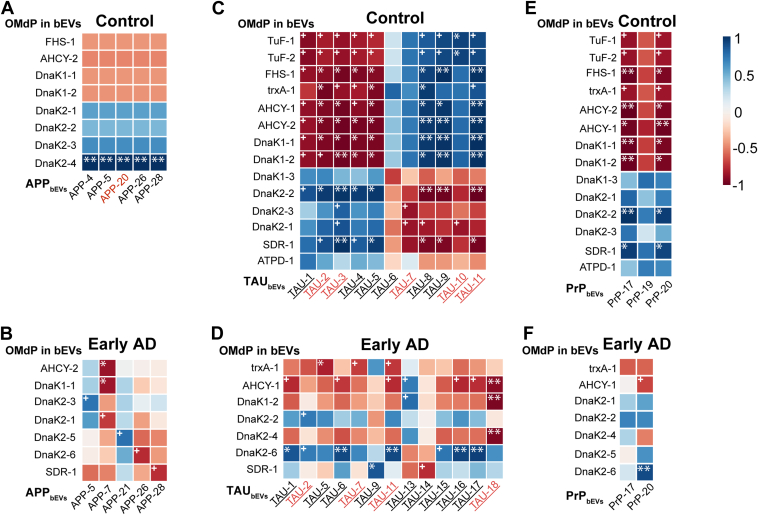
**Heatmap correlation plots including peptides from oral microbiome–derived proteins (OMdPs) in brain extracellular vesicles (bEVs) significantly modulated in Alzheimer’s disease (AD) or vascular dementia (VaD) and peptides from the AD-related proteins detected in bEVs.** The relative abundance of all detected peptides from the AD-related proteins amyloid precursor protein (APP), microtubule-associated protein TAU (TAU), presenilin 1 (PSEN1), and prion protein (PrP) in bEVs were considered in all these correlation analyses. Correlations were performed using the Pearson's correlation method based on the relative quantitation of every detected peptide. *R* values are represented as different degrees of color intensity with *blue shades* for positive correlations and *red shades* for negative correlations. *A* and *B*, heatmap correlations between peptides from OMdP in bEVs and APP in age-matched controls (control) and early AD (AD3), respectively. *C* and *D*, heatmap correlations between peptides from OMdP in bEVs and TAU in control and AD3, respectively. *E* and *F*, heatmap correlations between peptides from OMdP in bEVs and PrP in control and AD3, respectively. Peptides are codified according to the gene symbol of the protein followed by a number. Peptide sequences are detailed in Supplemental Tables S24 and S25. Peptide names in *red* indicate peptides that play a relevant role in AD based on the existent literature. Underlined TAU peptides indicate peptides that can be phosphorylated and therefore may play a role in the hyperphosphorylation of TAU that occurs during AD. All peptides reported have at least a correlation >0.5. + indicates significant correlations with a *p* ≤ 0.05; ∗ indicates significant correlations with a *p* ≤ 0.01; and ∗∗ indicates significant correlations with a *p* ≤ 0.001.

After conducting peptidome-wide correlation analyses, we aimed to determine if any significant relationships existed between OMdP and AD markers within the bEV fractions of dementia patients. Our findings revealed the presence of notable correlation patterns, which manifested as reversed correlations, affecting both age-matched controls and early AD subjects (Fig. 7). The research uncovered that many peptides derived from the OMdP in bEVs DnaK_1_ and DnaK_2_ exhibited positive correlations with the AD-associated protein APP in the bEVs of control subjects. Conversely, negative correlations were observed in early AD, as demonstrated in Figure 7, *A* and *B*. In addition, these peptides displayed similar patterns when compared with the AD marker TAU and exhibited a similar tendency to transition from positive to negative correlations with the protein PrP, as illustrated in Figure 7, *C*–*F*. The full set of peptide data obtained from these analyses, along with their corresponding sequences, is provided in Supplemental Tables S24 and S25.

### OMdP in WB Fractions Associate With AD Markers at the Peptide Level

**Fig. 8:**
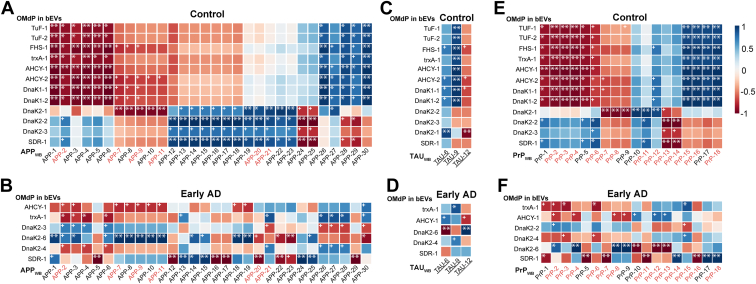
**Heatmap correlation plots including peptides from oral microbiome–derived proteins (OMdPs) in brain extracellular vesicles (bEVs) significantly modulated in Alzheimer’s disease (AD) or vascular dementia (VaD) and peptides from the AD-related proteins detected in the remaining whole brain (WB) proteome after isolation of bEVs.** The relative abundance of all detected peptides from the AD-related proteins amyloid precursor protein (APP), microtubule-associated protein TAU (TAU), presenilin 1 (PSEN1), and prion protein (PRP) in WB was considered in this correlation study. Correlations were performed using the Pearson's correlation method based on the relative quantitation of every detected peptide. *R* values are represented as different degrees of color intensity with *blue shades* for positive correlations and *red shades* for negative correlations. *A* and *B*, heatmap correlations between peptides from OMdP in bEVs and APP detected in whole brain (WB) (APP_WB_) in age-matched controls (control) and preclinical AD (AD3), respectively. *C* and *D*, heatmap correlations between peptides from OMdP in bEVs and TAU detected in WB (TAU_WB_) in control and AD3, respectively. *E* and *F*, heatmap correlations between peptides from OMdP in bEVs and PrP detected in WB (PrP_WB_) in control and AD3, respectively. Peptides are codified according to the gene symbol of the protein followed by a number. Peptide sequences are detailed in Supplemental Tables S24 and S26. Peptide names in *red* indicate peptides that play a relevant role in AD based on existent literature. Underlined TAU peptides indicate peptides that can be phosphorylated and therefore may play a role in the hyperphosphorylation of TAU that occurs during AD. All peptides reported have at least a correlation >0.5. + indicates significant correlations with a *p* ≤ 0.05; ∗ indicates significant correlations with a *p* ≤ 0.01; and ∗∗ indicates significant correlations with a *p* ≤ 0.001.

Reversed correlation patterns affecting the remaining WB fractions of subjects in the age-matched control and early AD groups were also identified involving specific peptides of the OMdP in bEVs DnaK_2_ and DnaK_3_ and the APP-20 and APP-21 (Fig. 8 and in Supplemental Tables S24 and S26). These results suggest that the presence of these peptides in WB fractions and bEVs is disrupted in early AD compared with controls.

### OMdP Differentially Circulates in Blood EVs of Clinical AD Subjects

To evaluate and verify the circulation capabilities of the identified OMdP in bEVs in the blood of AD patients, and in order to substantiate the results obtained, we scrutinized publicly available proteomics data from alternative clinical cohorts analyzing blood EVs from AD subjects. These confirmatory experiments revealed the presence of OMdP, DnaK, and ATPD in the circulating blood plasma EVs of subjects diagnosed with MCI, clinical AD, and age-matched controls, as presented in Table 1. Additional analysis noteworthily uncovered that the concentrations of OMdP DnaK in blood plasma EVs were markedly elevated in individuals with MCI and particularly in those with AD as compared with age-equivalent controls (Table 1).

**Table 1:** Identification of OMdPs in blood plasma circulating ECVs (OMdP in pEVs) from age-matched controls, subjects diagnosed with MCI, and clinical AD

Gene symbol	Protein description	Unique	Control	MCI	AD
DnaK	Chaperone protein DnaK	Yes	50 ± 11	65.74 ± 24∗	80.9 ± 17∗∗
ATPD	ATP synthase subunit beta	Yes	2.94 ± 2.1	2.89 ± 1.8	3.2 ± 1.7

Quantification of OMdP_pEVs_ is expressed as average ± SEM. OMdP in bEV protein identity was confirmed by the identification of unique peptides. Significant differences were assessed by one-way ANOVA with Bonferroni's correction for multiple comparisons. ∗ at MCI indicates significant differences at *p* ≤ 0.01 compared with control and significant differences at *p* ≤ 0.05 compared with AD, ∗∗ at AD indicates significant differences at *p* ≤ 0.01 compared with control and significant differences at *p* ≤ 0.05 compared with MCI.

### OM-EV Permeability Assays with BBB-oC

**Fig. 9:**
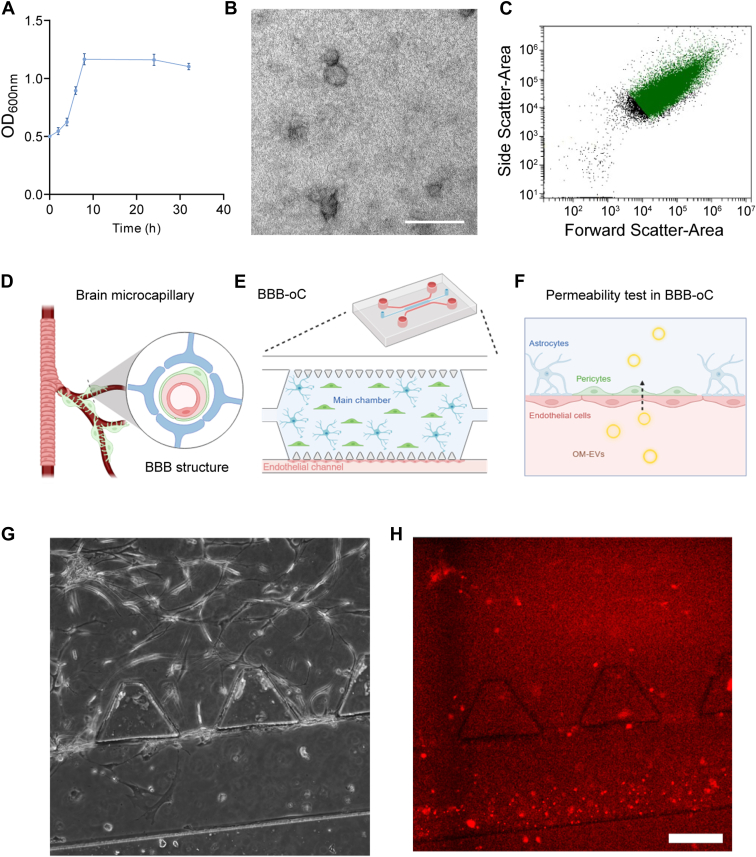
**Assessment of oral microbiome–derived extracellular vesicle (OM-EV) permeability using a BBB-on-a-chip (BBB-oC) model.***A*, growth curve of *Tannerella forsythia*, showing bacterial absorbance at 600 nm over time. *B*, representative transmission electron microscopy (TEM) micrographs of OM-EVs, confirming their vesicular morphology. The scale bars represent 100 nm. *C*, analysis of OM-EVs by flow cytometry analysis of OM-EVs. *D*, schematic representation of the brain microcapillary and blood–brain barrier (BBB) structure. *E*, illustration of the BBB-oC system, replicating key BBB components, including endothelial cells, astrocytes, and pericytes. *F*, schematic representation of the OM-EV permeability assay. *G* and *H*, bright-field and fluorescent microscopy images of the BBB-oC model showing labeled OM-EVs (*red*) that have crossed the BBB, demonstrating vesicle translocation across the barrier. The scale bars represent 100 nm. BBB, blood–brain barrier.

We selected *T. forsythia* as a representative oral microorganism among those identified in the metaproteomics study producing OMdP in bEVs. The growth curve of *T. forsythia* showed a rapid increase in an absorbance at 600 nm during the first 10 h of incubation, reaching the stationary phase at approximately 12 h, where it remained stable until the end of the incubation period (Fig. 9*A*). These results confirmed optimal bacterial growth under the defined anaerobic culture conditions. Following bacterial culture and OM-EV isolation, the morphological and biophysical characterization confirmed the successful isolation of vesicles, revealing an abundance of small, rounded vesicles (Fig. 9, *B* and *C*). To assess whether OM-EVs can traverse the BBB, and potentially reach the CNS, we used a BBB-oC model (Fig. 9, *D* and *E*). The BBB-oC permeability assay confirmed the ability of OM-EVs to cross the BBB under the tested conditions (Fig. 9, *H* and *I*). Fluorescent functionalized OM-EVs crossed the BBB with a relative permeability of ∼8.6 × 10^−5^ cm/s.

## Discussion

In recent years, there has been a heightened interest in investigating the impact of the microbiome on human health (65, 66), and alterations of the gut and OM have been specifically associated with neuropathological hallmarks (67, 68, 69). Nonetheless, it is crucial to deepen our comprehension of the potential role(s) of these microorganisms and their products in neurological diseases, particularly in relation to dementia-like conditions such as AD. This study aims to provide new insights into the multifactorial understanding of AD by presenting, to the best of our knowledge, the initial findings on how the products of certain OM-linked microorganisms reach the brain and their potential involvement in the pathophysiology of the disease.

Our study identified several key OMdP in bEVs that exhibit specific alterations throughout the progression of AD. Notable among these molecules are the HSPs DnaK, SDR, trxA, TuF, ligase (FHS), hydrolase (AHCY), and PstS. These bacterial proteins, particularly HSPs and SDR, play critical roles in maintaining homeostasis and mediating host–microbiota communication within the gut. HSPs are essential for protecting intestinal cells from stress and facilitating interactions between bacteria and host cells, thereby influencing immune responses and mucosal barrier integrity (70). The differential expression of these bacterial proteins along the colon correlates with the presence of specific health-associated and opportunistic microbes (71). Moreover, dysregulation of HSPs expression in the gut has been implicated in inflammatory diseases and associated with neurodegenerative conditions, indicating a potential impact on protein aggregation and neuroinflammation (72). Furthermore, these proteins were found to be associated with specific microorganisms of the OM. Specifically, this study revealed that protein products from *C*. *sakazakii* and *C. granulosum* are more prevalent in bEVs of age-matched controls and early AD subjects compared with those in advanced stages of AD. *C*. *sakazakii* has been associated with severe neonatal infections, including meningitis, by reaching the BBB through endocytosis of human brain microvascular endothelial cells. This process induces apoptosis, disrupts tight junctions, increases permeability, and triggers inflammation, leading to high mortality rates and adverse neurological effects in survivors (73). Similarly, and notably, *C. granulosum*, formerly known as *Cutibacterium acnes*, has been linked to AD-like pathology in animal models. Studies have shown that its inoculation in rats results in memory impairment and increased amyloid β deposits in the hippocampus, suggesting its involvement in neuroinflammation and neurodegeneration (74). While these findings provide insights into potential connections between these microorganisms and neurological conditions and contribute new insights into the “theory of the brain microbiome” (75), based on the emphasis on the protein products of these microorganisms rather than on the microorganisms themselves, the specific mechanisms by which their proteins interact with brain cells and contribute to AD pathology remain to be elucidated. Further research, based on the findings detailed herein, is necessary to elucidate the role of bEV encapsulation in this process and its potential protective or pathogenic effects in the context of AD progression.

The influence of aging on the microbiome must be acknowledged, as extensively reviewed by Jang *et al*. (76), especially regarding its impact on the gut. The aging process significantly alters microbial diversity and composition within the gut microbiome, contributing to various health issues. Research has demonstrated that as individuals age, there is a decline in beneficial bacteria, such as *Bifidobacteria* and *Lactobacilli*, accompanied by an increase in potentially pathogenic species, such as Enterobacteriaceae (76). The aging gut microbiome is characterized by reduced microbial richness and functional diversity, limiting the capacity of the microbiota to respond effectively to environmental changes. Similarly, aging significantly impacts the OM, leading to alterations in its composition and diversity, which can have broader health implications. A decline in bacterial diversity in the OM has been reported as individuals age, particularly after the age of 40, with a notable decrease in beneficial commensals (77). In addition, the presence of opportunistic pathogens increases with age, potentially contributing to systemic inflammation, a fact that may lead to increased risk of age-related diseases such as AD (78); thus far, very little is known regarding the effects of aging on the capacity of the OM to secrete OMdP in EVs. In addition, while previous studies have reported the effects of aging on BBB permeability, mediated by brain endothelial senescence (79), it remains unclear how increased BBB permeability may modulate the effects of OMdP in EVs, based on the findings reported here, and contribute to susceptibility to neurodegenerative conditions.

The present investigation also emphasizes the importance of examining alterations in peptides connected to dementia-associated diseases at the molecular level, rather than solely focusing on protein levels. Recent findings have demonstrated the significance of this approach, particularly concerning alterations in p-Tau and Tau levels in the human proteome (80, 81), which is further corroborated by the results observed in this study. Moreover, our findings elucidate that the presence of these dysbiotic-associated microorganism proteins exhibits variable associations with EVs and the brain parenchyma across the clinical progression of the disease. Notably, we identified robust negative correlations between the levels of OMdP in bEVs and the brain parenchyma in clinical AD cases involving DnaK, AHCY, and SDR. The aforementioned implications may suggest an impaired reduction of vesicle-mediated protein clearance through autophagy, a process previously reported to be impaired and associated with atypical compositions in bEVs in AD (44). This observation warrants further investigation. However, the revelation of OMdP related to bEVs, as well as the potential exchange of these molecules between bEVs and the brain parenchyma, which is demonstrated in this study and supported by other research (82, 83), prompted us to examine the possible relationships between OMdP and the key AD hallmark proteins that contribute to the proteinopathy of the disorder, including APP, TAU, and PSENs. The data from the *in silico* experiments performed have shown that OMdPs in bEVs are highly likely to interact with AD-related proteins, particularly those regions of the protein that are linked to the neuropathology of aging-associated dementias.

Several studies have investigated the potential involvement of the microorganism *Porphyromonas gingivalis* in the pathophysiology of AD. Recent research has indicated the potential involvement of this microorganism in neuroinflammatory processes of age-related dementias (84). Furthermore, Liu *et al*. (85) have recently proposed that outer membrane vesicles of this microorganism may traverse the BBB and reach the brain. Notwithstanding, our findings do not support this hypothesis. Similarly, Issilbeyeva *et al*. (86) have suggested that *P*. *gingivalis* may not play a central role in the development of AD in their search for new circulating biomarkers for the disease based on the analysis of the OM. These authors have identified specific discrepancies in the OM of individuals with AD in Central Asia. While cultural differences in dietary habits may impact the OM, these recent findings partially and remarkably align with our generated knowledge here. Thus, they propose the importance of identifying new biomarkers for the disease at the diagnostic and prognostic stages based on the molecular composition of the OM. Our findings here from an additional clinical cohort of subjects with MCI and AD indicate that OMdP in bEVs can be detected in blood plasma. Although these observations are preliminary, they provide valuable exploratory insights and generate hypotheses for future investigation into the potential systemic roles of OMdPs in cognitive decline and dementias.

Our study also provides additional insights into the permeability of OM-EVs across the BBB, as assessed using a BBB-oC model. While previous studies have proposed that bacterial EVs from the gut can impact neuroinflammation, particularly in the context of AD (87), our findings extend this concept to OM-EVs, highlighting their possible role in CNS molecular exchange. The ability of OM-EVs to cross the BBB supports the idea that oral dysbiosis could contribute to brain pathology through EV-mediated transport mechanisms. However, further studies will be needed to determine whether OMdP transported within these vesicles can interact with key neuropathological markers, such as APP, tau, and prion protein.

## Future Directions and Limitations

This work establishes a foundational proteomic profile of OM-EVs in AD and identifies potential interactions between their compositions and key neuropathological hallmark proteins. However, future research should focus on quantitatively validating the molecular changes identified, thereby extending the groundwork laid by this study. It is also important to characterize the underlying protein–protein interaction networks to further elucidate how OM-EVs engage with disease-related pathways.

We acknowledge that the relatively small cohort size in this discovery work, limited by the availability of specific post-mortem clinical samples, may introduce variability and restrict the generalizability of the findings, despite being supported by power calculations. Consequently, further validation in larger and independent cohorts is recommended as a future step to substantiate the findings.

In addition, although all extractions were performed with sterile reagents, sterile consumables, and under sterile conditions, the potential effects of reagents identified through mock extractions were not controlled for in this study, which may be considered a limitation. Similarly, a limited concordance between blood and brain tissues has been encountered and expected, potentially driven by cohort differences and the intrinsic challenges of blood analysis, which constrains interpretation; thus, our blood-based results should be considered exploratory.

## Data Availability

All data utilized in this study are publicly accessible, and no new MS data were generated. Data can be accessed *via* the specialized repository ProteomeXchange under the accession numbers PXD015578 for bEV proteomes and WB proteomes (31) and PXD024216 for plasma EV (47) proteomes. The scripts created for the bioinformatic analyses performed have also been made publicly available through GitHub: https://github.com/MariaMuletFernandez/OralMicrobiome.

## Supplemental Data

This article contains supplemental data.

## Ethical Approval

Informed consent was obtained from all subjects or their legal representatives at the respective biobanks that provided clinical samples for this study. The use of post-mortem brain tissues was performed in strict accordance with the Declaration of Helsinki and the Spanish Organic Law 3/5 December 2018 for Protection of Personal Data (LOPD). All experimental procedures were performed in accordance with institutional guidelines.

## Conflict of Interest

X. G. -P., A. S., M. M. and J. A. S. M. are inventors in the European patent application (PCT/EP2025/083064) filed by the nonprofit public research institutions: Biomedical Research Institute of Lleida (IRBLLEIDA) and the University of Lleida (UdL). The referred patent covers the identification and any potential application(s) of OMdP in BEVs as diagnostic and prognostic markers of age-related dementias. All other authors declare no competing interests.
