# Supplementary material for: Oral Microbiome–Derived Proteins in Brain Extracellular Vesicles Circulate and Tie to Specific Dysbiotic and Neuropathological Profiles in Age-Related Dementias
Source: Mol Cell Proteomics. 2025 Nov 17;24(12):101464. doi: 10.1016/j.mcpro.2025.101464 (PMC12757489; doi:10.1016/j.mcpro.2025.101464)
Supplement: Supplemental Materials [file mmc4.docx]

**Supplementary materials**

**Oral microbiome-derived proteins in brain extracellular vesicles circulate and tie to specific dysbiotic and neuropathological profiles in age-related dementias**

María Mulet, Jose Antonio Sánchez Milán^†^, Cristina Lorca^†^, María Fernández-Rhodes, Ana Adrados-Planell, María Consuelo Bejarano Castillo, Laura Saiz, María-Victoria Mateos-Moreno, Yoshiki Hase, Alex Mira, Alberto Rábano, Teodoro del Ser, Raj N. Kalaria, Anna Lagunas, Mònica Mir, Andrés Crespo, Josep Samitier, Xavier Gallart-Palau* and Aida Serra*

^†^José Antonio Sánchez Milán and Cristina Lorca contributed equally.

^*^ These authors share senior authorship and correspondence.

**Summary table:**

| ***Content*** | ***Page*** |
| --- | --- |
| ***Supplementary Figure 1.*** *Relative quantitation of MdP in bEVs commonly found in all the subjects of the study comparing between Gram-negatives and Gram-positives.* | *S-4* |
| ***Supplementary Figure 2.*** *Distribution of peptide intensities across the dataset.*  ***Supplementary Figure 3.*** *Representative MS/MS spectra of selected DnaK OM-derived peptides in brain-derived EVs.*  ***Supplementary Figure 4.*** *Representative MS/MS spectra of selected AHCY, TuF, FHS and trxA OM-derived peptides in brain-derived EVs.*  ***Supplementary Figure 5.*** *Representative MS/MS spectra of selected SDR, ATPD, ACAD, acnA and PstS OM-derived peptides in brain-derived EVs.*  ***Supplementary Figure 6.*** *Relative quantitation of OMdP in bEVs alongside the progression of AD comparing between Gram-negatives and Gram-positives.* | *S-5*  *S-6*  *S-7*  *S-8*  *S-9* |
| ***Supplementary Figure 7.*** *OMdP in bEVs detected in WB that do not show significant differences.*  ***Supplementary Figure 8.*** *Confidence scores of the negative control protein Chignolin.*  ***Supplementary Table 1.*** *Relevant available demographics of human post-mortem brains.*  ***Supplementary Table 2.*** *Relation of Gram-negative and Gram-positive microorganisms that potentially produce MdP in bEVs found in all groups of subjects.*  ***Supplementary Table 3.*** *Habitat and pathogenicity from the microorganisms that potentially produce MdP in bEVs found in all groups of subjects*  ***Supplementary Table 4.*** *Peptide intensities identified using the full and restricted databases for significant peptides across age-matched controls, AD3, and AD4 groups.*  ***Supplementary Table 5.*** *Protein coverage of confidently identified peptides using the entire and restricted databases across age-matched controls, AD3, and AD4 groups.*  ***Supplementary Table 6.*** *Additional peptides linked to restricted database validation.****Supplementary Table 7.*** *Cluster classification of DnaK protein accessions.*  ***Supplementary Table 8.*** *Summary of significant results from left-censored imputation and log2 transformation of spectral count data.*  ***Supplementary Table 9.*** *Summary of significant results from MaxLFQ-based analysis.*  ***Supplementary Table 10.*** *Habitat and pathogenicity from the microorganisms associated to MdP in bEVs that show significant modulation alongside the progression of AD.* | *S-10*  *S-11*  *S-12*  *Additional file*  *Additional file*  *Additional file*  *Additional file*  *Additional file*  *Additional file*  *Additional file*  *Additional file*  *Additional file* |
| ***Supplementary Table 11.*** *Relation of Gram-negative and Gram-positive microorganisms that potentially produce MdP in bEVs found significantly dysregulated linked to the progression of AD.*  ***Supplementary Table 12.*** *List of human homologous proteins of OMdP in bEVs, their associated functions and interacting proteins.* | *Additional file*  *Additional file* |
| ***Supplementary Table 13.*** *Human proteins that can potentially interact with OMdP in bEVs, their functions and cognitive decline-related disorders.*  ***Supplementary Table 14.*** *Interaction analysis between DnaK protein and human proteins associated to cognitive decline that interact with human homologous proteins of OMdP in bEVs.*  ***Supplementary Table 15.*** *Interaction analysis between AHCY protein and human proteins associated to cognitive decline that interact with human homologous proteins of OMdP in bEVs.*  ***Supplementary Table 16.*** *Interaction analysis between SDR protein and human proteins associated to cognitive decline that interact with human homologous proteins of OMdP in bEVs.*  ***Supplementary Table 17.*** *Interaction analysis between FHS protein and human proteins associated to cognitive decline that interact with human homologous proteins of OMdP in bEVs.*  ***Supplementary Table 18.*** *Interaction analysis between trxA protein and human proteins associated to cognitive decline that interact with human homologous proteins of OMdP in bEVs.*  ***Supplementary Table 19.*** *Interaction analysis between acnA protein and human proteins associated to cognitive decline that interact with human homologous proteins of OMdP in bEVs.*  ***Supplementary Table 20.*** *Interaction analysis between ACAD protein and human proteins associated to cognitive decline that interact with human homologous proteins of OMdP in bEVs.*  ***Supplementary Table 21.*** *Interaction analysis between PstS protein and human proteins associated to cognitive decline that interact with human homologous proteins of OMdP in bEVs.*  ***Supplementary Table 22.*** *Interaction analysis between TuF protein and human proteins associated to cognitive decline that interact with human homologous proteins of OMdP in bEVs.*  ***Supplementary Table 23.*** *Interaction analysis between ATPD protein and human proteins associated to cognitive decline that interact with human homologous proteins of OMdP in bEVs.*  ***Supplementary Table 24.*** *Codification of peptides from OMdP in bEVs detected in bEVs by LC-MS/MS used in Figures 7 and 8.*  ***Supplementary Table 25.*** *Codification of peptides from human AD-related proteins found in bEVs detected by LC-MS/MS used in Figure 7.*  ***Supplementary Table 26.*** *Codification of peptides from human AD-related proteins found in WB detected by LC-MS/MS used in Figure 8.*  ***Supplementary References*** | *Additional file*  *Additional file*  *Additional file*  *Additional file*  *Additional file*  *Additional file*  *Additional file*  *Additional file*  *Additional file*  *Additional file*  *Additional file*  *Additional file*  *Additional file*  *Additional file*  *S-13* |
| ***Supplementary Dataset 1.*** *Supplementary_dataset_BEVs_Dem_R1_vF.* | *Additional file* |
| ***Supplementary Dataset 2****. Supplementary_dataset_WB_Dem_R1_vF.* | *Additional file* |

**Supplementary Figure 1.** Relative quantitation of MdP in bEVs commonly found in all the subjects of the study comparing between Gram-negatives and Gram-positives.

**
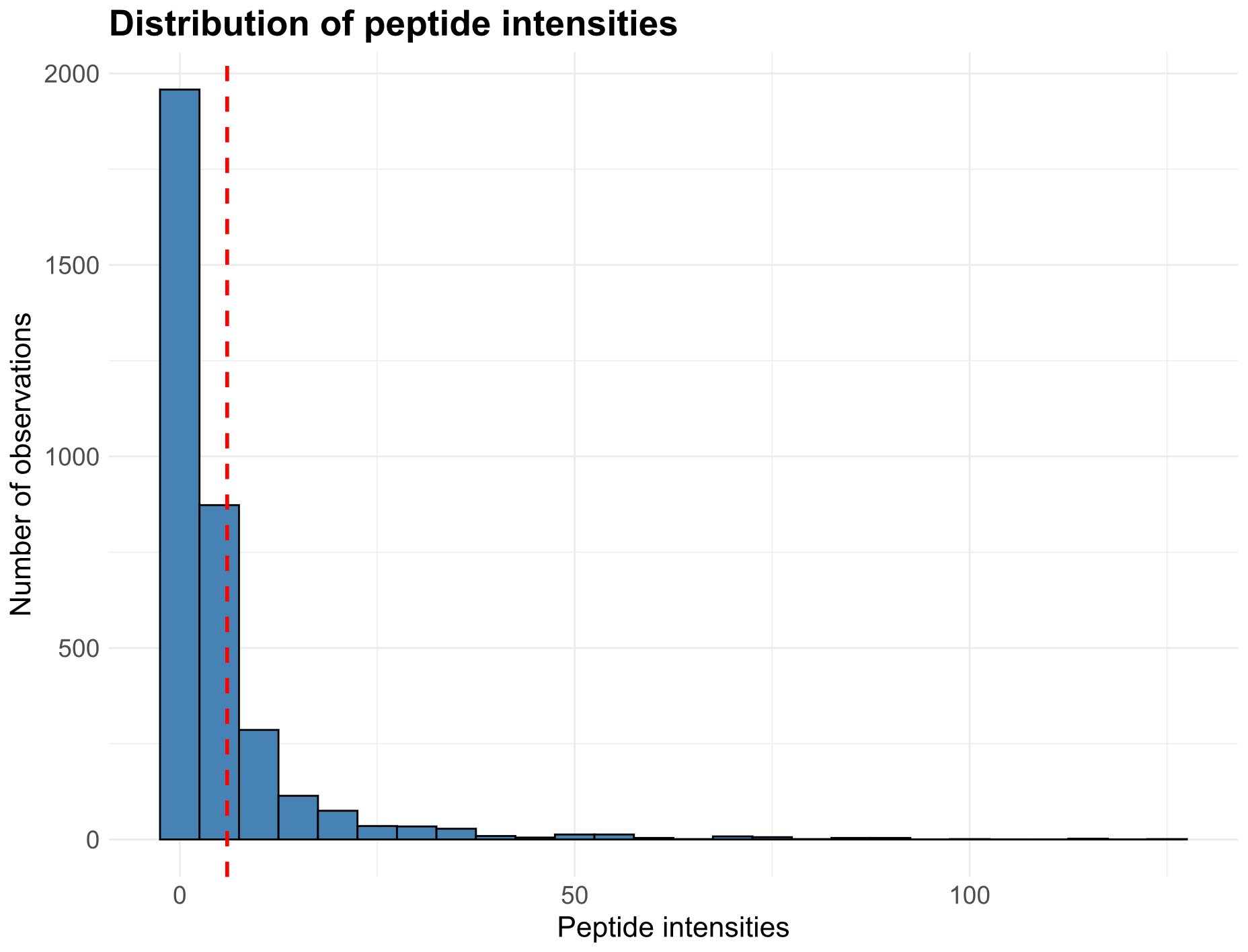
**

**Supplementary Figure 2.** Distribution of peptide intensities. The histogram presents the distribution of peptide intensities, categorized into intervals of 5, as identified across all LC-MS/MS experiments conducted. The dashed line represents the 80th percentile threshold, below which all peptides included in our analyses are situated (spectral count intensity ≤ 6).

**
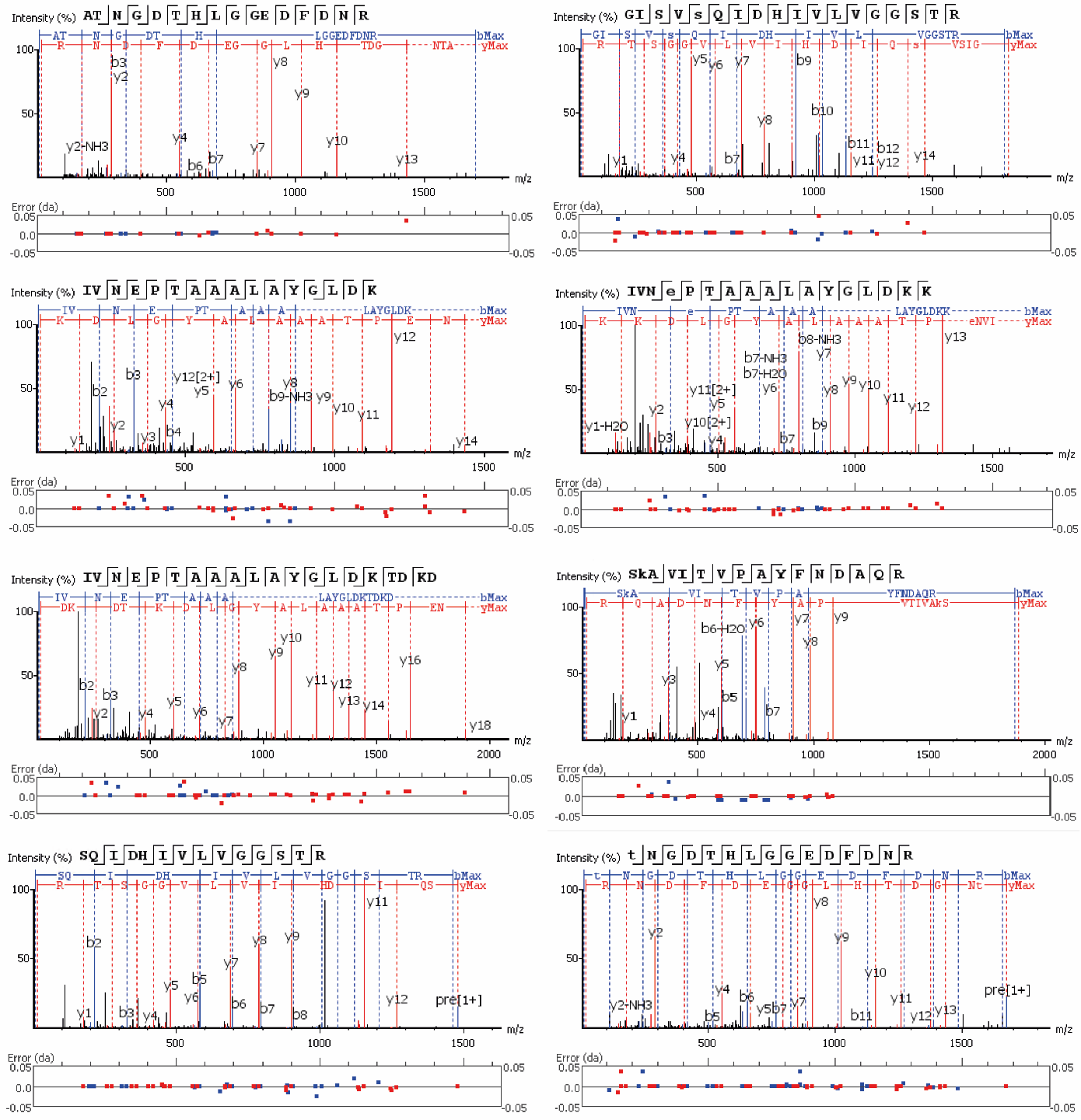
Supplementary Figure 3.** Representative MS/MS spectra of selected DnaK OM-derived peptides identified in brain-derived EVs.

**
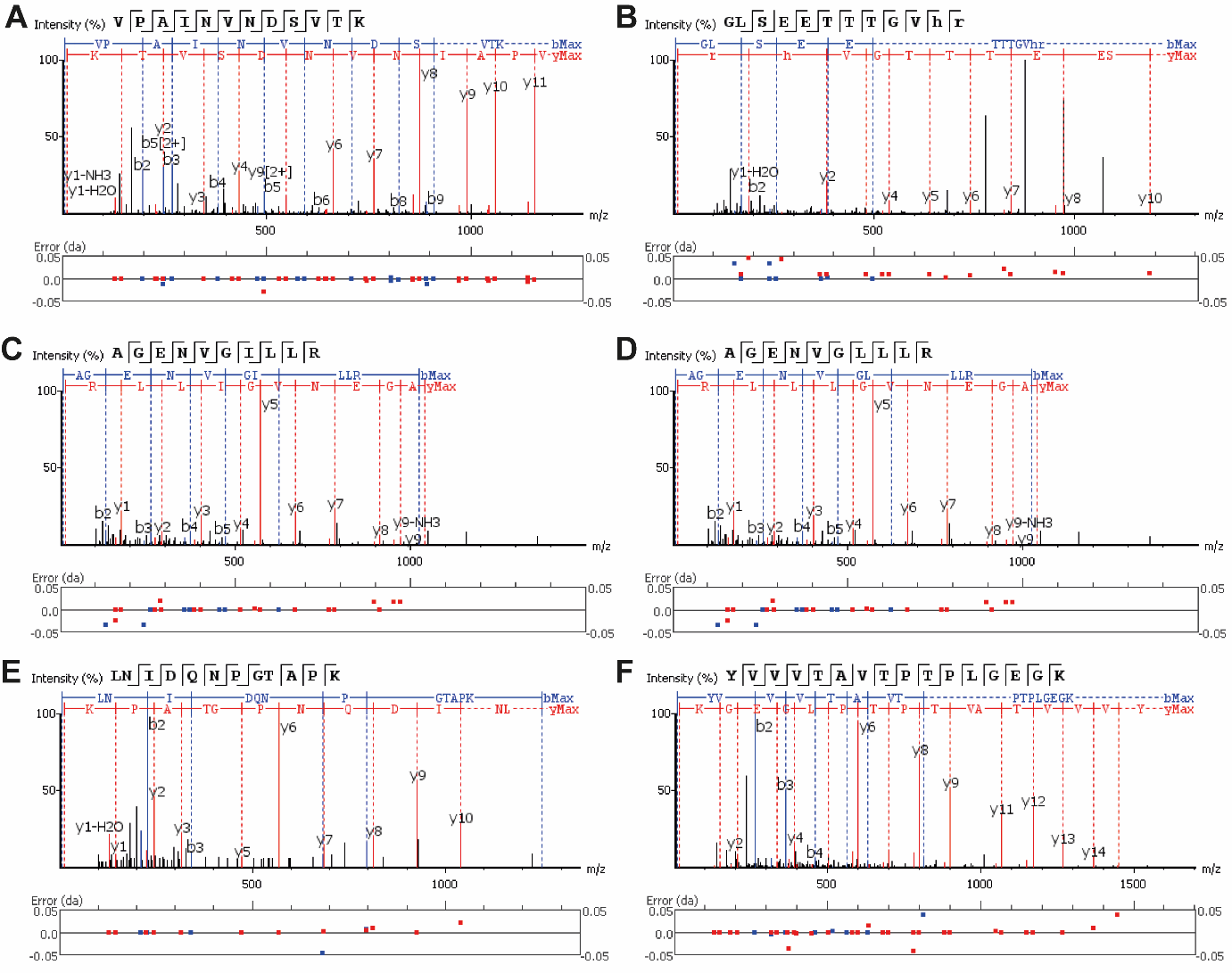
**

**Supplementary Figure 4.** Representative MS/MS spectra of selected AHCY, TuF, FHS and trxA OM-derived peptides in brain-derived EVs. **A, B.** AHCY; **C, D**. TuF; **E**. trxA; **F**. FHS.

**
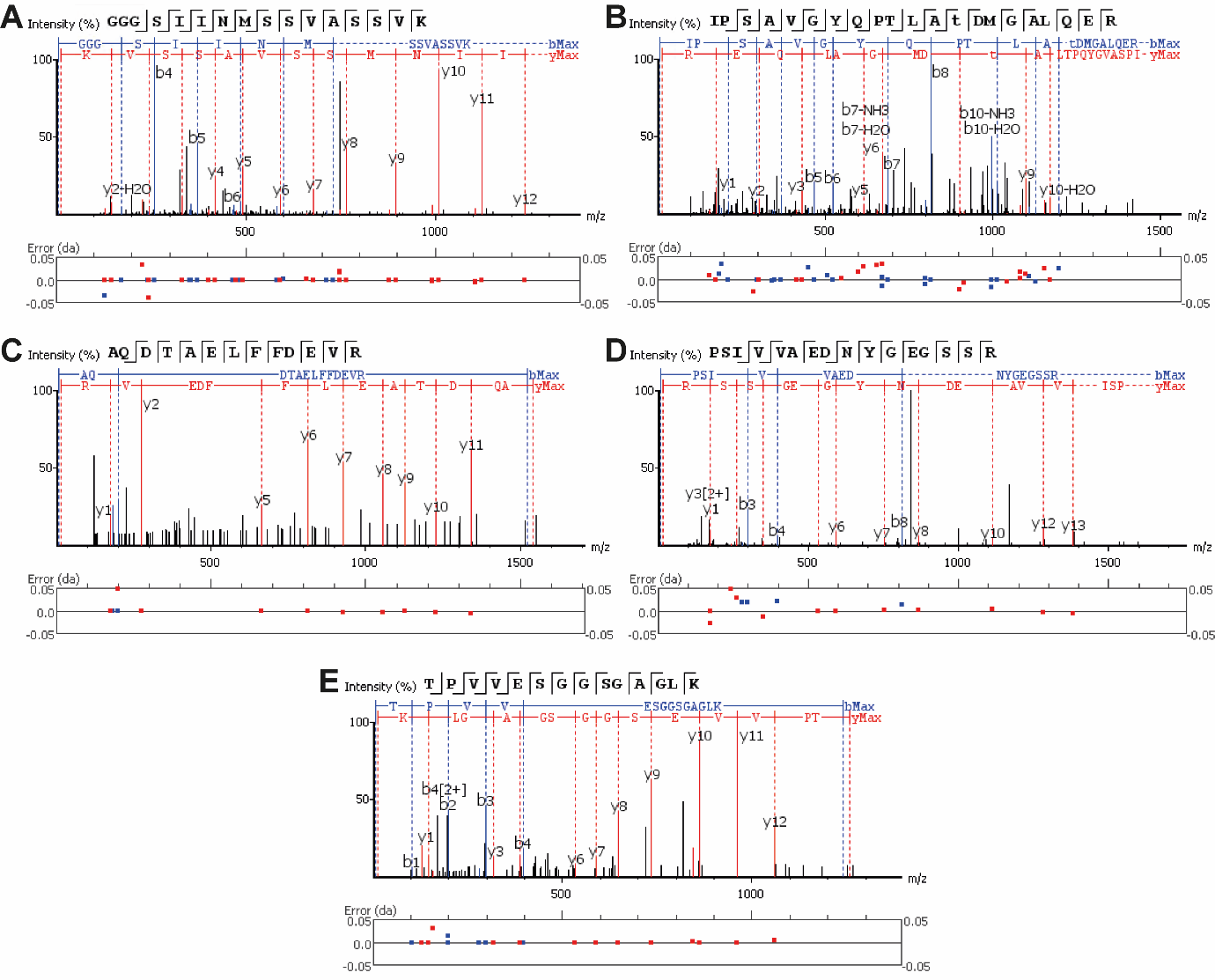
**

**Supplementary Figure 5.** Representative MS/MS spectra of selected SDR, ATPD, ACAD, acnA and PstS OM-derived peptides in brain-derived EVs. **A.** SDR; **B.** ATPD; **C.** ACAD; **D**. acnA; **E**. PstS.

**Supplementary Figure 6.** Relative quantitation of OMdP in bEVs alongside the progression of AD comparing between Gram-negatives and Gram-positives.

**D**

**A**

**E**

**B**

**C**

**AD3**

**AD4**

**AD5**

**AD6**

**VaD**

**0**

**2×10**

**5**

**4×10**

**5**

**6×10**

**5**

**8×10**

**5**

**1×10**

**6**

**TuF**

**Study groups**

**Protein intensity**

#

#

**F**

**C**

**Supplementary Figure 7.** Protein levels of OMdP in bEVs detected in WB proteome of age-matched controls (C), AD subjects (AD3 to AD6) and VaD subjects. Protein intensity expressed as area under the curve (AUC). Significance was assessed by parametric two-way ANOVA with Bonferroni’s correction or by Fisher’s two-way ANOVA or by non-parametric Kruskal Wallis with a minimum significance level *p* ≤ .05. + indicates significant differences determined by ANOVA with Fisher’s LSD p ≤ .05, ++ indicates significant differences determined by ANOVA with Fisher’s LSD p ≤ .01, # indicates significant differences determined by nonparametric one-way ANOVA on ranks p ≤ .05 and ## indicates significant differences determined by nonparametric one-way ANOVA on ranks p ≤ .01.

**Supplementary Figure 8.** Chignolin was used as a negative control for the docking simulation performed with the software HDOCK. Plot of confidence scores obtained in the docking simulations performed between the negative control protein Chignolin and the OMdP in bEVs. Docking simulations were performed using the PDB of each OMdP in bEVs as follows: DnaK from *A. actinomycetecomitans*, AHCY from *M. aeruginosavorus*, SDR from *P. fluorescens*, FHS from *C. granulosum*, trxA from *E. coli*, acnA from *P. dentalis*, ACAD from *R. picketti*, PstS from *A. tumefaciens*, TuF from *A. propionica* and ATPD from *R. capsulatus*. The red dotted line indicates the confidence score threshold of 0.7.

|  | **Age** | **Male/Female** | ***PMD** |
| --- | --- | --- | --- |
| **Controls** | 68 ± 3 | 7/5 | 22 ± 3 |
| **AD3/Early AD** | 74 ± 3 | 5/8 | 24 ± 3 |
| **AD4/Intermediate AD** | 83 ± 9 | 2/5 | 15 ± 5 |
| **AD5/Advanced AD** | 69 ± 5 | 4/2 | 17 ± 5 |
| **AD6/Final stage AD** | 69 ± 4 | 4/2 | 13 ± 6 |
| **VaD** | 67 ± 6 | 3/0 | 17 ± 12 |

**Supplementary table 1.** Relevant available demographics of human post-mortem brains. *PMD refers to post-mortem delay expressed in hours.

**Supplementary references**

1. Brenner DJ, Hollis DG, Moss CW, English CK, Hall GS, Vincent J, et al. Proposal of *Afipia* *Gen-Nov*, with *Afipia-Felis* *Sp-Nov* (Formerly the Cat Scratch Disease Bacillus), *Afipia-Clevelandensis* *Sp-Nov* (Formerly the Cleveland-Clinic-Foundation Strain), *Afipia-Broomeae* *Sp-Nov*, and 3 Unnamed Genospecies. J Clin Microbiol. 1991;29(11):2450-60.

2. Aleti G, Baker JL, Tang X, Alvarez R, Dinis M, Tran NC, et al. Identification of the bacterial biosynthetic gene clusters of the oral microbiome illuminates the unexplored social language of bacteria during health and disease. mBio. 2019;10(2).

3. Fine DH, Schreiner H, Velusamy SK. *Aggregatibacter*, a low abundance pathobiont that influences biogeography, microbial dysbiosis, and host defense capabilities in periodontitis: The history of a bug, and localization of disease. Pathogens. 2020;9(3);179.

4. Sood S. Infective Endocarditis by *Aggregatibacter paraphrophilus*: Case report and literature review. J Clin Diagn Res. 2013;7(11):2577-8;.

5. Broa AL, Cruz I, Cotrim C, Diogo J. *Aggregatibacter aphrophilus* aortic valve infective endocarditis. Infectio. 2016.

6. Szafranski SP, Kilian M, Yang I, Bei der Wieden G, Winkel A, Hegermann J, et al. Diversity patterns of bacteriophages infecting *Aggregatibacter* and *Haemophilus* species across clades and niches. ISME J. 2019;13(10):2500-22.

7. Cosseau C, Romano-Bertrand S, Duplan H, Lucas O, Ingrassia I, Pigasse C, et al. *Proteobacteria* from the human skin microbiota: Species-level diversity and hypotheses. One Health. 2016;2:33-41.

8. Adnan M, Khan S, Patel M, Al-Shammari E, Ashankyty IMA. *Agrobacterium*: a potent human pathogen. Rev Med Microbiol. 2013;24(4):94-7.

9. Kumar PS, Griffen AL, Barton JA, Paster BJ, Moeschberger ML, Leys EJ. New bacterial species associated with chronic periodontitis. J Dent Res. 2003;82(5):338-44.

10. Devresse A, Labriola L, Dossin T, Vatlet M, Hantson P. *Atopobium rimae* bacteremia complicated by an infection-related glomerulonephritis in a cardiac transplanted patient. Transpl Infect Dis. 2016;18(4):637-8.

11. Colombo AP, Boches SK, Cotton SL, Goodson JM, Kent R, Haffajee AD, et al. Comparisons of subgingival microbial profiles of refractory periodontitis, severe periodontitis, and periodontal health using the human oral microbe identification microarray. J Periodontol. 2009;80(9):1421-32.

12. Genco RJ, LaMonte MJ, McSkimming DI, Buck MJ, Li L, Hovey KM, et al. The subgingival microbiome relationship to periodontal disease in older women. J Dent Res. 2019;98(9):975-84.

13. Tresse O. Campylobacter In: Colgrave ML, editor. Proteomics in food Science: From farm to fork. London: Academic Press; 2017. p. 449-67.

14. Perez-Chaparro PJ, Goncalves C, Figueiredo LC, Faveri M, Lobao E, Tamashiro N, et al. Newly identified pathogens associated with periodontitis: a systematic review. J Dent Res. 2014;93(9):846-58.

15. Liu F, Ma R, Wang Y, Zhang L. The Clinical Importance of *Campylobacter concisus* and other human hosted *Campylobacter* Species. Front Cell Infect Microbiol. 2018;8:243.

16. Elston MJ, Dupaix JP, Opanova MI, Atkinson RE. *Cutibacterium acnes* (formerly *Proprionibacterium acnes*) and shoulder surgery. Hawaii J Health Soc Welf. 2019;78(11 Suppl 2):3-5.

17. Choi HA, Ahn SO, Lim HD, Kim GJ. Growth suppression of a gingivitis and skin pathogen *Cutibacterium* (*Propionibacterium*) *acnes* by medicinal plant extracts. Antibiotics (Basel). 2021;10(9).

18. Downes J, Munson M, Wade WG. *Dialister invisus* *sp. nov*., isolated from the human oral cavity. Int J Syst Evol Microbiol. 2003;53(Pt 6):1937-40.

19. O'Neil CR, Wilson E, Missaghi B. Bone and joint infections due to *Haemophilus parainfluenzae*: Case report and review of the literature. Can J Infect Dis Med. 2016;2016;4503025.

20. Boucher MB, Bedotto M, Couderc C, Gomez C, Reynaud-Gaubert M, Drancourt M. *Haemophilus pittmaniae* respiratory infection in a patient with siderosis: a case report. J Med Case Rep. 2012;6:120.

21. Carretero RG, Dominguez MR, Bastian MR, Lomba ML. *Lactobacillus salivarius* infection as a postoperative complication after bariatric surgery. Enferm Infec Micr Cl. 2018;36(1):60-1.

22. Kabore WAD, Dembele R, Bagre TS, Konate A, Boisrame S, Chevalier V, et al. Characterization and antimicrobial susceptibility of *Lactococcus lactis* isolated from endodontic infections in Ouagadougou, Burkina Faso. Dent J (Basel). 2018;6(4).

23. Dashiff A, Junka RA, Libera M, Kadouri DE. Predation of human pathogens by the predatory bacteria *Micavibrio aeruginosavorus* and *Bdellovibrio bacteriovorus*. J Appl Microbiol. 2011;110(2):431-44.

24. Jacob Raja SA, Raja JJ, Vijayashree R, Priya BM, Anusuya GS, Ravishankar P. Evaluation of oral and periodontal status of leprosy patients in Dindigul district. J Pharm Bioallied Sci. 2016;8(Suppl 1):S119-S21.

25. Macovei L, McCafferty J, Chen T, Teles F, Hasturk H, Paster BJ, et al. The hidden 'mycobacteriome' of the human healthy oral cavity and upper respiratory tract. J Oral Microbiol. 2015;7:26094.

26. Javali MA, Patil V, Ayesha H. Periodontal disease as the initial oral manifestation of abdominal tuberculosis. Dent Res J (Isfahan). 2012;9(5):634-7.

27. Ryan MP, Pembroke JT. The Genus *Ochrobactrum* as major opportunistic pathogens. Microorganisms. 2020;8(11);1797.

28. Mobley HLT. *Proteus mirabilis* overview. Methods Mol Biol. 2019;2021:1-4.

29. Ardila Medina CM. Efecto de las enterobacterias en pacientes con periodontitis crónica. Avances en Periodoncia e Implantología Oral. 2010;22:27-35.

30. Bor B, Bedree JK, Shi W, McLean JS, He X. *Saccharibacteria* *(TM7)* in the Human Oral Microbiome. J Dent Res. 2019;98(5):500-9.

31. Dashper SG, Mitchell HL, Le Cao KA, Carpenter L, Gussy MG, Calache H, et al. Temporal development of the oral microbiome and prediction of early childhood caries. Sci Rep. 2019;9(1):19732.

32. Friedman EM, Valdez TA. Chapter 18 - Otitis externa. In: Feigin RD, Cherry JD, Demmler-Harrison GJ, Kaplan SL, editors. Feigin and Cherry's textbook of pediatric infectious diseases (Sixth Edition). Philadelphia: W.B. Saunders; 2009. p. 212-6.

33. dos Santos BR, Demeda CF, da Silva EE, de Britto MH, Lima KC, de Melo MC. Prevalence of subgingival S*taphylococcus* at periodontally healthy and diseased sites. Braz Dent J. 2014;25(4):271-6.

34. Yu IS, Yeom SJ, Kim HJ, Lee JK, Kim YH, Oh DK. Substrate specificity of S*tenotrophomonas nitritireducens* in the hydroxylation of unsaturated fatty acid. Appl Microbiol Biotechnol. 2008;78(1):157-63.

35. Lenartova M, Tesinska B, Janatova T, Hrebicek O, Mysak J, Janata J, et al. The oral microbiome in periodontal Health. Front Cell Infect Microbiol. 2021;11:629723.

36. Handley P, Coykendall A, Beighton D, Hardie JM, Whiley RA. *Streptococcus crista sp. nov.*, a viridans streptococcus with tufted fibrils, isolated from the human oral cavity and throat. Int J Syst Bacteriol. 1991;41(4):543-7.

37. Abranches J, Zeng L, Kajfasz JK, Palmer SR, Chakraborty B, Wen ZT, et al. Biology of oral *Streptococci*. Microbiol Spectr. 2018;6(5).

38. Burton JP, Wescombe PA, Moore CJ, Chilcott CN, Tagg JR. Safety assessment of the oral cavity probiotic *Streptococcus salivarius K12*. Appl Environ Microbiol. 2006;72(4):3050-3.

39. Ansbro K, Wade WG, Stafford GP. *Tannerella serpentiformis sp. nov.,* isolated from the human mouth. Int J Syst Evol Microbiol. 2020;70(6):3749-54.

40. Colombo APV, Tanner ACR. The role of bacterial biofilms in dental caries and periodontal and peri-implant diseases: a historical perspective. J Dent Res. 2019;98(4):373-85.

41. Beall CJ, Campbell AG, Griffen AL, Podar M, Leys EJ. Genomics of the uncultivated, periodontitis-associated bacterium *Tannerella sp. BU045 (Oral Taxon 808)*. mSystems. 2018;3(3).

42. Renvoise A, Raoult D, Roux V. *Actinomyces massiliensis sp. nov.*, isolated from a patient blood culture. Int J Syst Evol Microbiol. 2009;59(Pt 3):540-4.

43. van Houte J, Lopman J, Kent R. The final pH of bacteria comprising the predominant flora on sound and carious human root and enamel surfaces. J Dent Res. 1996;75(4):1008-14.

44. Li J, Li Y, Zhou Y, Wang C, Wu B, Wan J. Actinomyces and alimentary tract diseases: a review of its biological functions and pathology. Biomed Res Int. 2018;2018:3820215.

45. Broa AL. *Aggregatibacter aphrophilus* aortic valve infective endocarditis. Elsevier. 2016;110:3.

46. Bowden GHW. *Actinomyces*, *Propionibacterium propionicus*, and *Streptomyces*. In: Baron S, editor. Medical Microbiology. Galveston (TX); 1996.

47. Jeong JH, Kweon OJ, Kim HR, Kim TH, Ha SM, Lee MK. A novel species of the genus *Arsenicicoccus* isolated from human blood using whole-genome sequencing. Ann Lab Med. 2021;41(3):323-7.

48. Almeida-da-Silva CLC, Alpagot T, Zhu Y, Lee SS, Roberts BP, Hung SC, et al. *Chlamydia pneumoniae* is present in the dental plaque of periodontitis patients and stimulates an inflammatory response in gingival epithelial cells. Microb Cell. 2019;6(4):197-208.

49. Welch JLM, Rossetti BJ, Rieken CW, Dewhirst FE, Borisy GG. Biogeography of a human oral microbiome at the micron scale. P Natl Acad Sci USA. 2016;113(6):E791-E800.

50. Sokol-Leszczynska B, Leszczynski P, Lachowicz D, Rostkowska O, Niemczyk M, Piecha T, et al. *Corynebacterium coyleae* as potential urinary tract pathogen. Eur J Clin Microbiol. 2019;38(7):1339-42.

51. Gosney MA, Martin MV, Wright AE, Gallagher M. *Enterobacter sakazakii* in the mouths of stroke patients and its association with aspiration pneumonia. Eur J Intern Med. 2006;17(3):185-8.

52. Tellez Corral MA, Herrera Daza E, Cuervo Jimenez HK, Bravo Becerra MDM, Villamil JC, Hidalgo Martinez P, et al. Cryptic oral microbiota: what is its role as obstructive sleep apnea-related periodontal pathogens? Int J Environ Res Public Health. 2023;20(3).

53. Costa C, Merino-Ribas A, Ferreira C, Campos C, Silva N, Pereira L, et al. Characterization of oral *Enterobacteriaceae* prevalence and resistance profile in chronic kidney disease patients undergoing peritoneal dialysis. Front Microbiol. 2021;12:736685.

54. Chung JH, Jeong H, Ryu CM. Complete genome sequences of *Enterobacter cancerogenus* *CR-Eb1* and *Enterococcus sp. strain CR-Ec1*, isolated from the larval gut of the greater wax moth, galleria mellonella. Genome Announc. 2018;6(7).

55. Zaatout N. Presence of non-oral bacteria in the oral cavity. Arch Microbiol. 2021;203(6):2747-60.

56. Komiyama EY, Lepesqueur LS, Yassuda CG, Samaranayake LP, Parahitiyawa NB, Balducci I, et al. *Enterococcus* Species in the oral cavity: prevalence, virulence factors and antimicrobial susceptibility. PLoS One. 2016;11(9):e0163001.

57. Kanazuru T, Sato EF, Nagata K, Matsui H, Watanabe K, Kasahara E, et al. Role of hydrogen generation by *Klebsiella pneumoniae* in the oral cavity. J Microbiol. 2010;48(6):778-83.

58. Zawadzki PJ, Perkowski K, Padzik M, Mierzwinska-Nastalska E, Szaflik JP, Conn DB, et al. Examination of oral microbiota diversity in adults and older adults as an approach to prevent spread of risk factors for human infections. Biomed Res Int. 2017;2017:8106491.

59. Fernandez-Natal MI, Saez-Nieto JA, Medina-Pascual MJ, Valdezate-Ramos S, Guerra-Laso JM, Rodriguez-Pollan RH, et al. First report of bacteremia by *Janibacter terrae* in humans. Infection. 2015;43(1):103-6.

60. Kitamoto S, Nagao-Kitamoto H, Jiao Y, Gillilland MG, 3rd, Hayashi A, Imai J, et al. The intermucosal connection between the mouth and gut in commensal pathobiont-driven colitis. Cell. 2020;182(2):447-62 e14.

61. De Champs C, Rich C, Chandezon P, Chanal C, Sirot D, Forestier C. Factors associated with antimicrobial resistance among clinical isolates of *Klebsiella pneumoniae*: 1-year survey in a French university hospital. Eur J Clin Microbiol Infect Dis. 2004;23(6):456-62.

62. Gaiser RA, Halimi A, Alkharaan H, Lu L, Davanian H, Healy K, et al. Enrichment of oral microbiota in early cystic precursors to invasive pancreatic cancer. Gut. 2019;68(12):2186-94.

63. Szczerba I, Krzeminski Z. [Occurrence of bacteria in the mouth from genera of *Micrococcus*, *Kocuria*, *Nesterenkonia*, *Kytococcus* and *Dermacoccus*]. Med Dosw Mikrobiol. 2002;54(1):29-34.

64. Levenga H, Donnelly P, Blijlevens N, Verweij P, Shirango H, de Pauw B. Fatal hemorrhagic pneumonia caused by infection due to *Kytococcus sedentarius*: a pathogen or passenger? Ann Hematol. 2004;83(7):447-9.

65. Badet C, Thebaud NB. Ecology of lactobacilli in the oral cavity: a review of literature. Open Microbiol J. 2008;2:38-48.

66. Oshima K, Hayashi J, Toh H, Nakano A, Shindo C, Komiya K, et al. Complete genome sequence of *Parascardovia denticolens* JCM 12538T, Isolated from Human Dental Caries. Genome Announc. 2015;3(3).

67. Kononen E, Fteita D, Gursoy UK, Gursoy M. *Prevotella* species as oral residents and infectious agents with potential impact on systemic conditions. J Oral Microbiol. 2022;14(1):2079814.

68. Medina CMA. Effect of enterobacteriaceae in patients with chronic periodontitis. Avances en periodoncia e implantología oral. 2010;22:27-36.

69. Souto R, Silva-Boghossian CM, Colombo AP. Prevalence of *Pseudomonas aeruginosa* and *Acinetobacter spp*. in subgingival biofilm and saliva of subjects with chronic periodontal infection. Braz J Microbiol. 2014;45(2):495-501.

70. Scales BS, Dickson RP, LiPuma JJ, Huffnagle GB. Microbiology, genomics, and clinical significance of the *Pseudomonas fluorescens* species complex, an unappreciated colonizer of humans. Clin Microbiol Rev. 2014;27(4):927-48.

71. Conti S, dos Santos SS, Koga-Ito CY, Jorge AO. *Enterobacteriaceae* and *pseudomonadaceae* on the dorsum of the human tongue. J Appl Oral Sci. 2009;17(5):375-80.

72. Cicek M, Hascelik G, Mustak HK, Diker KS, Sener B. [Accurate diagnosis of *Pseudomonas luteola* in routine microbiology laboratory: on the occasion of two isolates]. Mikrobiyol Bul. 2016;50(4):621-4.

73. Sun X, Li M, Xia L, Fang Z, Yu S, Gao J, et al. Alteration of salivary microbiome in periodontitis with or without type-2 diabetes mellitus and metformin treatment. Sci Rep. 2020;10(1):15363.

74. Vinokurov MG, Yurinskaya MM, Grachev SV, Prokhorenko IR. Lipopolysaccharide from *Rhodobacter capsulatus* suppresses the effect of endotoxins from various *E. coli* chemotypes on the priming and apoptosis of human neutrophils. Dokl Biochem Biophys. 2009;424:35-7.

75. Oshima K, Hayashi J, Toh H, Nakano A, Omori E, Hattori Y, et al. Complete Genome Sequence of *Scardovia inopinata JCM 12537T*, Isolated from Human Dental Caries. Genome Announc. 2015;3(3).

76. Kressirer CA, Smith DJ, King WF, Dobeck JM, Starr JR, Tanner ACR. *Scardovia wiggsiae* and its potential role as a caries pathogen. J Oral Biosci. 2017;59(3):135-41.

77. LaMonte MJ, Genco RJ, Buck MJ, McSkimming DI, Li L, Hovey KM, et al. Composition and diversity of the subgingival microbiome and its relationship with age in postmenopausal women: an epidemiologic investigation. BMC Oral Health. 2019;19(1):246.

78. Gobbetti M. *STREPTOCOCCUS* | Introduction. In: Batt CA, Tortorello ML, editors. Encyclopedia of Food Microbiology. London: Academic Press; 2014. p. 535-553.

79. Roux E, Nicolas A, Valence F, Siekaniec G, Chuat V, Nicolas J, et al. The genomic basis of the *Streptococcus thermophilus* health-promoting properties. BMC Genomics. 2022;23(1):210.

80. Higashi S, Moore DJ, Minegishi M, Kasanuki K, Fujishiro H, Kabuta T, et al. Localization of MAP1-LC3 in vulnerable neurons and lewy bodies in brains of patients with dementia with lewy bodies. J Neuropath Exp Neur. 2011;70(4):264-80.

81. Suh J, Romano DM, Nitschke L, Herrick SP, DiMarzio BA, Dzhala V, et al. Loss of Ataxin-1 potentiates alzheimer's pathogenesis by elevating cerebral BACE1 transcription. Cell. 2019;178(5):1159-1175.

82. Ling DJ, Song HJ, Garza D, Neufeld TP, Salvaterra PM. Abeta42-induced neurodegeneration via an age-dependent autophagic-lysosomal injury in *Drosophila*. Plos One. 2009;4(1);e4201.

83. Underwood R, Wang B, Carico C, Whitaker RH, Placzek WJ, Yacoubian TA. The GTPase Rab27b regulates the release, autophagic clearance, and toxicity of alpha-synuclein. J Biol Chem. 2020;295(23):8005-16.

84. Zhao Y, Ho P, Yih Y, Chen C, Lee WL, Tan EK. LRRK2 variant associated with Alzheimer's disease. Neurobiol Aging. 2011;32(11):1990-3.

85. Aidaralieva NJ, Kamino K, Kimura R, Yamamoto M, Morihara T, Kazui H, et al. Dynamin 2 gene is a novel susceptibility gene for late-onset Alzheimer disease in non-APOE-epsilon 4 carriers. J Hum Genet. 2008;53(4):296-302.

86. Dharshini SAP, Taguchi YH, Gromiha MM. Exploring the selective vulnerability in Alzheimer disease using tissue specific variant analysis. Genomics. 2019;111(4):936-49.

87. Zhao BX, Shan Y, Yang Y, Yu ZL, Li TF, Wang XF, et al. Transcriptome-wide association analysis of brain structures yields insights into pleiotropy with complex neuropsychiatric traits. Nat Commun. 2021;12(1);2878.

88. Stevanin G, Fujigasaki H, Lebre AS, Camuzat A, Jeannequin C, Dode C, et al. Huntington's disease-like phenotype due to trinucleotide repeat expansions in the TBP and JPH3 genes. Brain. 2003;126:1599-603;.

89. Passarella D, Ciampi S, Di Liberto V, Zuccarini M, Ronci M, Medoro A, et al. Low-density lipoprotein receptor-related protein 8 at the crossroad between cancer and neurodegeneration. Int J Mol Sci. 2022;23(16);8921.

90. Roy ER, Wang BP, Wan YW, Chiu G, Cole A, Yin ZR, et al. Type I interferon response drives neuroinflammation and synapse loss in Alzheimer disease. J Clin Invest. 2020;130(4):1912-30.

91. Linders PTA, van der Horst C, ter Beest M, van den Bogaart G. Stx5-mediated ER-golgi transport in mammals and yeast. Cells-Basel. 2019;8(8);780.

92. Cervellati C, Valacchi G, Tisato V, Zuliani G, Marsillach J. Evaluating the link between Paraoxonase-1 levels and Alzheimer's disease development. Minerva Med. 2019;110(3):238-50.

93. Saeidi M, Shakeri R, Marjani A, Khajeniazi S. Alzheimer's disease and Paraoxonase 1 (PON1) gene polymorphisms. Open Biochem J. 2017;11:47-55.

94. Theus MH, Brickler T, Meza AL, Coutermarsh-Ott S, Hazy A, Gris D, et al. Loss of NLRX1 exacerbates neural tissue damage and NF-kappaB signaling following brain injury. J Immunol. 2017;199(10):3547-58.

95. Buscham TJ, Eichel-Vogel MA, Steyer AM, Jahn O, Strenzke N, Dardawal R, et al. Progressive axonopathy when oligodendrocytes lack the myelin protein CMTM5. Elife. 2022;11;e75523.

96. Haenig C, Atias N, Taylor AK, Mazza A, Schaefer MH, Russ J, et al. Interactome mapping provides a network of neurodegenerative disease proteins and uncovers widespread protein aggregation in affected brains. Cell Rep. 2020;32(7);108050.

97. Steinacker P, Aitken A, Otto M. 14-3-3 proteins in neurodegeneration. Semin Cell Dev Biol. 2011;22(7):696-704.

98. Drache B, Diehl GE, Beyreuther K, Perlmutter LS, Konig G. Bcl-xl-specific antibody labels activated microglia associated with Alzheimer's disease and other pathological states. J Neurosci Res. 1997;47(1):98-108.

99. Kuhlmann T, Glas M, zum Bruch C, Mueller W, Weber A, Zipp F, et al. Investigation of bax, bcl-2, bcl-x and p53 gene polymorphisms in multiple sclerosis. J Neuroimmunol. 2002;129(1-2):154-60.

100. Reynolds CA, Hong MG, Eriksson UK, Blennow K, Wiklund F, Johansson B, et al. Analysis of lipid pathway genes indicates association of sequence variation near SREBF1/TOM1L2/ATPAF2 with dementia risk. Hum Mol Genet. 2010;19(10):2068-78.

101. Pulina MV, Hopkins M, Haroutunian V, Greengard P, Bustos V. C99 selectively accumulates in vulnerable neurons in Alzheimer's disease. Alzheimers Dement. 2020;16(2):273-82.

102. Matrone C, Iannuzzi F, Annunziato L. The Y(682)ENPTY(687) motif of APP: Progress and insights toward a targeted therapy for Alzheimer's disease patients. Ageing Res Rev. 2019;52:120-8.

103. Iannuzzi F, Sirabella R, Canu N, Maier TJ, Annunziato L, Matrone C. Fyn tyrosine kinase elicits amyloid precursor protein Tyr682 phosphorylation in neurons from Alzheimer's disease patients. Cells-Basel. 2020;9(8).

104. Goedert M, Spillantini MG. A century of Alzheimer's disease. Science. 2006;314(5800):777-81.

105. Hanger DP, Anderton BH, Noble W. Tau phosphorylation: the therapeutic challenge for neurodegenerative disease. Trends Mol Med. 2009;15(3):112-9.

106. Brandt R, Trushina NI, Bakota L. Much more than a cytoskeletal protein: Physiological and pathological functions of the non-microtubule binding region of Tau. Front Neurol. 2020;11:590059.

107. Wang Y, Mandelkow E. Tau in physiology and pathology. Nat Rev Neurosci. 2016;17(1):5-21.

108. Bergen Mv. Assembly of τ protein into Alzheimer paired helical filaments depends on a local sequence motif (306VQIVYK311) forming β structure. Proceedings of the National Academy of Sciences. 2000;97:5129-34.

109. Zhang Y, Zhao Y, Zhang L, Yu W, Wang Y, Chang W. Cellular prion protein as a receptor of toxic amyloid-beta42 oligomers is important for Alzheimer's disease. Front Cell Neurosci. 2019;13:339.

110. Chen S, Yadav SP, Surewicz WK. Interaction between human prion protein and amyloid-beta (Abeta) oligomers: role OF N-terminal residues. J Biol Chem. 2010;285(34):26377-83.
